# A Unique Type of Highly-Activated Microglia Evoking Brain Inflammation via Mif/Cd74 Signaling Axis in Aged Mice

**DOI:** 10.14336/AD.2021.0520

**Published:** 2021-12-01

**Authors:** Chenghao Jin, Yijie Shao, Xiaotao Zhang, Jiani Xiang, Ruize Zhang, Zeyu Sun, Shuhao Mei, Jingyi Zhou, Jianmin Zhang, Ligen Shi

**Affiliations:** ^1^Department of Neurosurgery, Second Affiliated Hospital, School of Medicine, Zhejiang University, Hangzhou, China.; ^2^Brain Research Institute, Zhejiang University, Hangzhou, Zhejiang, China.; ^3^Collaborative Innovation Center for Brain Science, Zhejiang University, Hangzhou, Zhejiang, China.

**Keywords:** microglia, brain inflammation, aged mice, Cd74, single cell RNA sequencing

## Abstract

Senescence-associated alterations of microglia have only recently been appreciated in the aged brain. Although our previous study has reported chronic inflammation in aged microglia, the mechanism remains poorly understood. Here, we performed morphological detection and transcriptomic analysis of aged microglia at the single cell level. Aged mice showed a large quantity and a large body volume of microglia in the brain. Six subgroups of microglia with unique function were identified by single cell RNA sequencing. Three out of six subgroups showed dramatic variations in microglia between aged and young mice. A unique type of highly-activated microglia (HAM) was observed in aged mice only, with specific expression of several markers, including Lpl, Lgals3, Cst7, and Cd74. Gene clusters with functional implications in cell survival, energy metabolism, and immuno-inflammatory responses were markedly activated in HAM. Mechanistically, neuron-released Mif, acting through Cd74 receptor in HAM, promoted the immunochemotactic activity of microglia, which then triggered immuno-inflammatory responses in aged brains. These findings may reveal new targets for reducing age-related brain inflammation to maintain brain health.

Microglia are the primary resident immune cells of the central nervous system (CNS) and constitute 5-12% of brain cells [1]. Microglia participate in phagocytosis and the elimination of debris, immune cell recruitment, synaptic pruning, and neurogenesis in a physiological manner [2]. Moreover, microglia possess highly specialized plasticity with the capability to adjust its functional phenotypes according to the local CNS environments [3]. Emerging evidence indicates that aging markedly triggers whole-brain inflammation, which contributes to the activation of microglia in the homeostatic brain [4]. Recently, several lines of evidence suggest these age-associated changes in microglia are notably observed in Alzheimer’s diseases and in multiple sclerosis [5, 6]. Thus, unraveling the functional alterations of aged microglia is fundamentally important to prevent brain aging.

Accumulating evidence implicates genome-wide transcriptional changes of microglia in aged brains of both humans and rodents [7-11]. Microglia have been reported to be involved in the priming state associated with the inflammatory environment in aged mice [7]. Our previous study also reported that aged microglia up-regulated a variety of biological processes, including regulation of cell adhesion, cytokine production, leukocyte migration, and regulation of defense response in the homeostatic brain [10]. Similar age-associated changes involved in immune-inflammatory response were also notably observed in human microglia [8, 9]. However, not all the microglia in the aged brain were in this unique priming state [12, 13]. Indeed, using single cell RNA sequencing (scRNA-seq) and cytometry by time-of-flight (CyTOF), only a small group of microglia was identified to be highly activated during aging [12, 13]. It remains poorly understood how this specific age-related subgroup of microglia is activated, and whether aging has an important influence on other microglia. In addition, a recent study revealed that T cell infiltration of aged mouse brains led to decreased activation of neural stem cells [14]. However, it remains to be determined whether recruitment of infiltrated T cells is related to aged microglia.

In the current study, morphological alterations in aged microglia prompted us to closely investigate functional changes in microglia during aging. Three out of six microglial subgroups showed dramatic alterations in aged mice compared to young mice. A unique type of highly-activated microglia (HAM) were observed only in aged mice, in which specific expression of several markers were observed, including Lpl (encoding lipoprotein lipase), Lgals3 (encoding galectin-3), Cst7 (encoding cystatin-F), and Cd74 (encoding H-2 class II histocompatibility antigen γ chain). Gene clusters with functional implications in cell survival, energy metabolism, and immuno-inflammatory responses were markedly activated in HAM. Mechanistically, neuron-released macrophage migration inhibitory factor (Mif) acts through the Cd74 receptor in HAM to promote immunochemotactic activity of microglia, which triggers immuno-inflammatory responses in the aged brain. These findings may create a new therapeutic avenue for anti-aging and for preventing age-related neurodegenerative disorders.

## MATERIALS AND METHODS

### Animals

Young male C57BL/6 mice (8-10 weeks old) were purchased from SLAC Laboratory Animal Company Limited (Shanghai, China). Aged male C57BL/6 mice (18 months old) were obtained from Beijing Vital River Laboratory Animal Technology Co. Ltd (Beijing, China). Mice were housed in a temperature and humidity-controlled animal facility with a 12-h light/dark cycle. The animal protocol was approved by the Institutional Ethics Committee of the Second Affiliated Hospital, Zhejiang University School of Medicine. The procedures were conducted according to the National Institutes of Health's Guide for the Care and the Use of Laboratory Animals and the ARRIVE (Animal Research: Reporting in vivo Experiments) guidelines. All efforts were made to minimize animal suffering and the number of animals used.

### Immunofluorescence of brain sections

Mice were anesthetized and then perfused intracardially with 0.1M phosphate-buffered saline (PBS), followed by perfusion with 4% paraformaldehyde. The whole brain was immersed in 4% paraformaldehyde for 24 h, followed by dehydration in serial 15% and 30% sucrose solutions. The brain samples were then cut into coronal slices (25-μm thick). After preprocessing with 5% BSA and 0.3% triton X-100, the sections were incubated at 4°C overnight with the following primary antibodies: anti-Iba1 (Abcam, ab5076), anti-NeuN (Abcam, ab104224), anti-MIF (Abcam, ab7207), anti-Lipoprotein lipase (Abcam, ab21356), and anti-Galectin 3 (Abcam, ab76245). Then, the sections were incubated with donkey secondary antibody conjugated with Alexa Fluor 488 or 594 (1:1000, Invitrogen) at room temprature for 1 h and washed three times with PBS with Tween-20 PBST. Slides were mounted and coverslipped with Fluoromount-G containing DAPI (Yeasen Biotech), and then sections were observed and analyzed using an Olympus fluorescence microscope.

### Imaris 3D rendering

The software Imaris (Bitplane, v.9.0) was used to reconstruct 3D images of immunofluorescence signal for quantification of the volume of ionized calcium-binding adapter molecule 1 (Iba-1)^+^ cells. Briefly, image stacks obtained by confocal microscopy were imported into Imaris, and the surface module was used to generate 3D structures of each color channel. After a region of interest was selected, the absolute intensity of each source channel was used for reconstruction. Smoothing was set to 0.568 μm for all channels and images. To differentiate the target signal from background, a threshold was set to remove the non-specific signals. After the 3D-rendered images were reconstructed, the volume of Iba-1 immunofluorescence was calculated automatically. All images were processed with the same adjustments and parameters.

### Preprocessing and clustering analysis of scRNA-seq data

We took advantage of the following two scRNA-seq datasets from the gene expression omnibus (GEO) database: 1) the expression matrix of whole brain in young and aged mice (GSE129788), and 2) the expression matrix of the subventricular zone (SVZ) in young and aged mice (PRJNA450425).

Basic processing of the scRNA-seq data was performed with the Seurat package (v.3.1.0) in R (v.3.6.1). After reading the original dataset, we applied the following criteria for quality control (QC) to filter out low-quality cells: 1) number of expressed genes was less than 200 or more than 2500, and 2) the percentage of mitochondrial RNA was greater than 10%. Then, we performed unsupervised clustering analysis on scRNA-seq data. Briefly, gene counts for cells that passed QC were normalized to the total expression and log-transformed. Then, highly variable genes were detected using the FindVariableFeatures function with default parameters. Linear scaling was then applied, and the mitochondrial contamination was removed using the ScaleData function. Principal component analysis was performed on the scaled data for dimensional reduction. Clusters were identified using the FindClusters function. Non-linear dimensional reduction methods including uniform manifold approximation and projection (UMAP) and t-distributed stochastic neighbor embedding (t-SNE), were used to visualize clustering results.

### Differential expression analysis

Differentially expressed genes were found using the FindAllMarkers (or FindMarkers) function which ran Wilcoxon rank sum tests. Differentially expressed genes (DEGs) were defined as genes with a natural log fold change > 0.25 or < -0.25, and with a Bonferroni adjusted P-value < 0.05.

### Functional enrichment analysis

Functional enrichment analysis was performed with the online tool Metascape (http://metascape.org). All genes in the mouse genome were used as the enrichment background. After a list of DEGs was submitted, Metascape returned a list of significantly overrepresented (P < 0.01) ontology terms with a minimum count of 3, and an enrichment factor (the ratio between the observed counts and the counts expected by chance) larger than 1.5. Terms were grouped into clusters based on their membership similarities. The activation z-score of each ontology term was calculated by the R package GOplot as (number of upregulated genes - number of downregulated genes) / square root of the number of genes assigned to a term. A term was predicted to be significantly activated with a z-score ≥ 2 and a P-value < 0.01; a term was predicted to be significantly inhibited with a z-score ≤ -2 and a P-value < 0.01. Enrichment data were visualized as bubble plots or chord plots using GOplot.

### Pseudotime ordering analysis

Monocle R package (v.2.12.0) was applied for pseudotime ordering analysis of scRNA-seq data from aged microglia. Briefly, the top 500 high variable genes were selected as ordering genes. Discriminative dimensionality reduction with the trees (DDRTree) algorithm was applied to reduce the dimensionality of data. Then, cellular trajectory was generated and visualized using orderCells and plot_cell_trajectory functions, respectively. After the starting point of the cellular trajectory was chosen, the pseudotemporal order of microglia was generated. Gene expression pattern was profiled in this pseudotemporal order using FateID R package (v.0.1.9).

### Cell-cell interaction analysis

To illustrate communication between microglia and other cells, the iTALK R package (v.0.1.0) was applied. Briefly, a built-in human ligand-receptor database was converted to homologue genes in mice based on Ensembl (http://www.ensembl.org/). After the top 10% highly expressed genes in each cluster were extracted, these genes were mapped to the previously mentioned ligand-receptor database to find potential ligand-receptor pairs. Matched ligand-receptor pairs were visualized using the LRPlot function.

### Statistical analysis

Single-cell RNA-seq data were analyzed as described above. Other datasets are presented as mean ± standard deviation (SD). Statistical comparison of the means between two groups was accomplished by the Student’s t test or the Mann-Whitney U test (both two-tailed). The analyses were performed using SPSS(v23) software. A P-value less than or equal to 0.05 was deemed statistically significant.

## RESULTS

### Distinct alterations in morphological and transcriptomic features of microglia in response to aging

**Figure 1.**
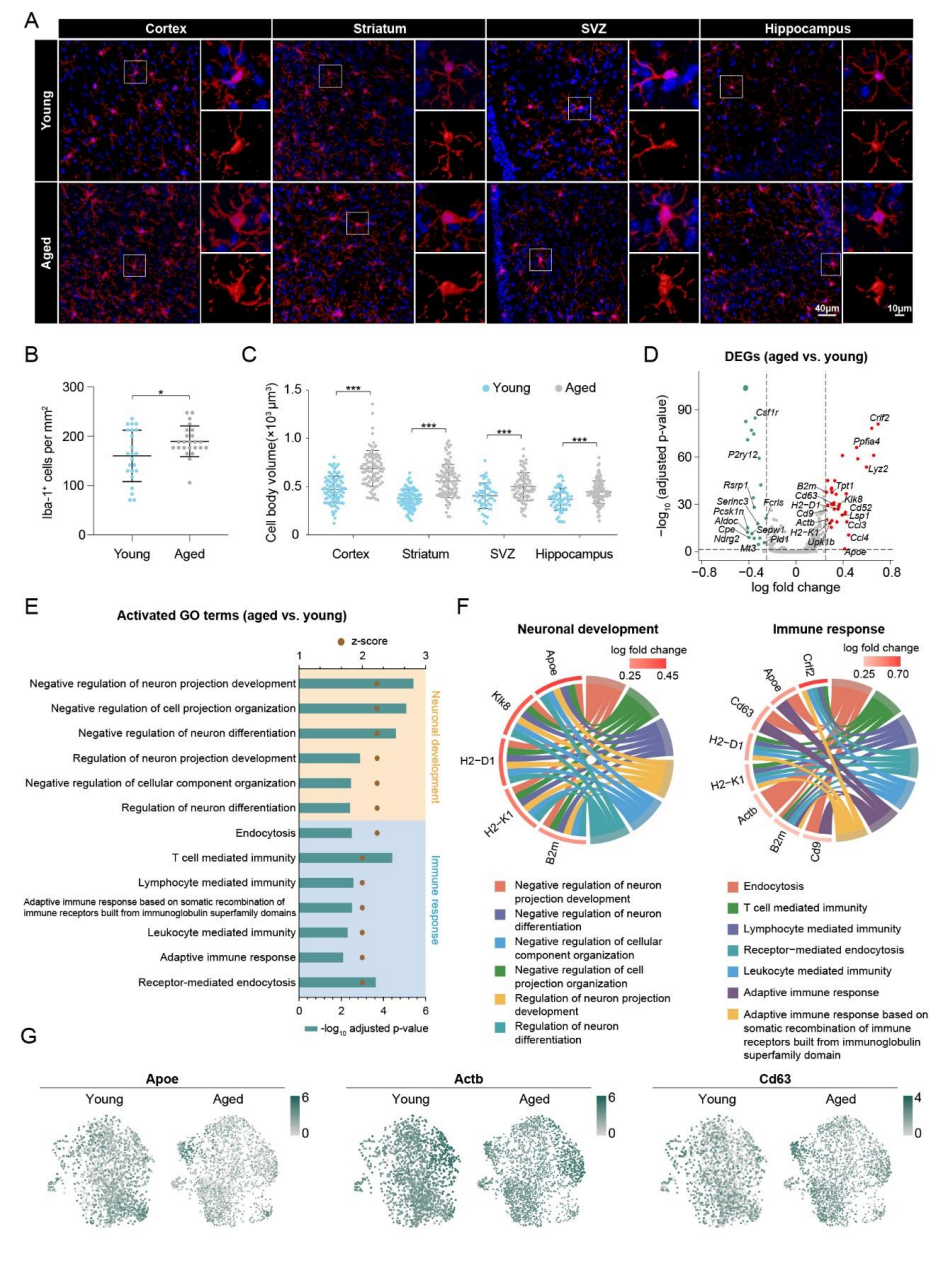
Morphologic and functional differences between microglia from young and aged mice. (A) Representative images showing the immunofluorescence signal of Iba-1+ cells in four different brain regions (cortex, striatum, SVZ, and hippocampus, from left to right). Cells were counterstained with DAPI for nuclear labeling. Squares: regions that were zoomed-in (upper right) and 3D-rendered by Imaris (lower right). *P < 0.05, **P < 0.01, ***P < 0.001, aged vs. young. Scale bars: 40μm for low power; 10μm for high power. (B) The density of Iba-1+ cells was quantified. n = 23 vs. 24 sections for young vs. aged mice, respectively, from four brain regions (cortex, striatum, SVZ, hippocampus). (C) The body volume of Iba-1+ cells was quantified. n = 98 vs. 89, 103 vs. 102, 53 vs. 82 and 65 vs. 96 cells for cortex, striatum, SVZ and hippocampus in young vs. aged mice, respectively. (D) A volcano plot depicts the results of differential expression analysis in aged microglia compared to young microglia. Differentially expressed genes (DEGs; log fold change > 0.25 or < -0.25, Bonferroni adjusted P-value < 0.05) are colored (red for upregulated DEGs and green for downregulated DEGs). Non-ribosomal and non-mitochondrial DEGs are annotated. (E) GO enrichment analysis was performed using Metascape on the DEGs from (D). The activation status of enriched terms was assessed by calculating their activation z-scores using GOplot. Shown are the 13 GO terms that were predicted to be activated (z-score ≥ 2) with age. These terms were divided into two functional clusters (neuronal development and immune response), according to the corresponding biologic functions. (F) The relationship between the activated GO terms in (E) and the involved genes is depicted. A circos plot shows genes related to neuronal development(left) and immune response(right) functional clusters, which were explored for their involvement in six and seven functional sub-categories, respectively. (G) Expression profiles of three representative genes involved in neuronal development and immune response genes in both young and aged microglia are shown using a uniform manifold approximation and projection (UMAP) visualization approach.

Aging has been reported to have different effects on multiple aspects of microglia. To examine the morphologic differences between aged and young microglia in mice, we performed immunofluorescence staining with specific microglia marker Iba-1 on four different brain regions (i.e., cortex, striatum, hippocampus and SVZ) in aged and young mice (Fig. 1A). A significant increase (18.45%) in microglial number was observed in aged mice (Fig. 1B). Cell body volume quantified by Imaris showed a significant increase in aged microglia compared with young microglia (Fig. 1C). These findings were consistent with the results from previous studies in both human and rodents [15-17], which sparked our curiosity to explore the transcriptomic changes of microglia in response to aging.

The large-scale dataset (GSE129788) provides us a resource for further investigating the transcriptomic changes in aged microglia [18]. On the basis of quality control, 37069 cells from the whole brains of eight young and eight old mice were used for unsupervised clustering analysis, and the microglial cluster containing 3853 cells was extracted for sub-clustering and further downstream analysis (Supplementary Fig. 1A). Differential expression analysis was then performed to explore the transcriptomic differences between aged and young microglia. The aged microglia highly express ribosomal genes and lowly express mitochondrial genes (Supplementary Fig. 1B). Except for these two classes of genes, 17 upregulated and 12 downregulated genes (1333 detected genes) were observed in aged microglia compared with young microglia (Fig. 1D and Supplementary Table 1). Gene ontology (GO) enrichment analysis of these 29 significantly changed genes suggested that in aged microglia, the immune response was evoked, whereas regulation of neuron development, differentiation, and projection organization was suppressed (Fig. 1E). We further displayed the relationship between GO terms and significantly changed genes on chord plots (Fig. 1F). Five key genes, Apoe (encoding apolipoprotein E), Klk8 (encoding kallikrein-8), H2-D1 (encoding H-2 class I histocompatibility antigen, D-B α chain), H2-K1 (encoding H-2 class I histocompatibility antigen, K-B α chain), and B2m (encoding β-2-microglobulin), were identified in all GO terms regarding negative regulation of neuron development, differentiation, and projection organization (Fig. 1F). Eight key genes, Crlf2 (encoding cytokine receptor-like factor 2), Apoe, Cd63 (encoding CD63 antigen), H2-D1, H2-K1, Actb (encoding β-actin), B2m, and Cd9 (encoding CD9 antigen), were involved in immune responses including endocytosis, T cell-mediated immunity, lymphocyte-mediated endocytosis, adaptive immune response, and more (Fig. 1F). Interestingly, the above-mentioned genes showed a remarkable heterogeneity in gene expression of microglia in both young and aged mice (Fig. 1G, Supplementary Fig. 1C).

### Three out of six microglial subsets showed dramatic alterations in aged mice

**Figure 2.**
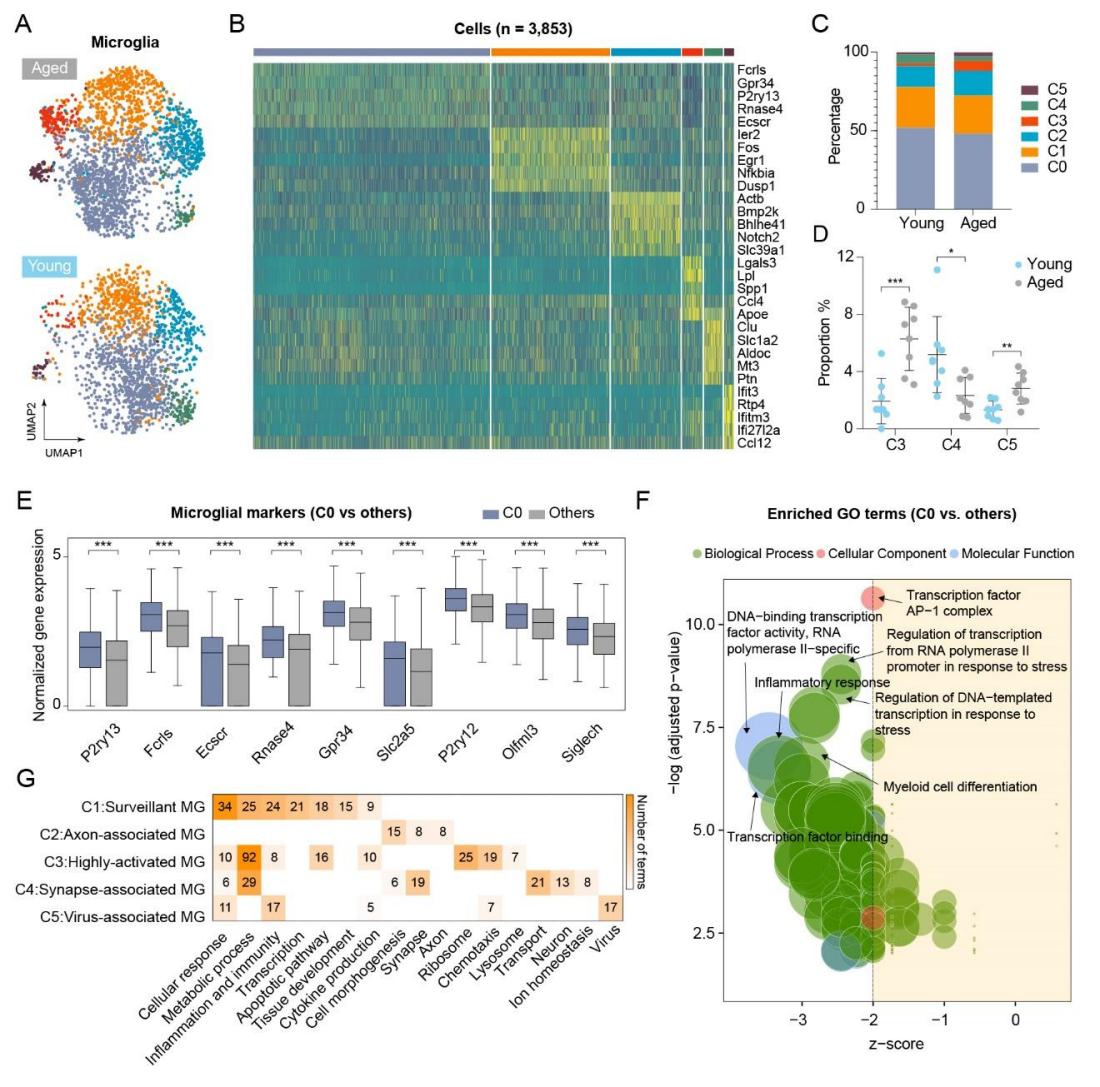
Three of six distinct microglia subclusters show dramatic alterations in aged mice. (A) Microglia were divided into six subclusters (C0 to C5) after unsupervised clustering analysis using Seurat R package. UMAP shows the six microglia subclusters grouped by age. (B) Heatmap visualization of expression level for the top five marker genes in each microglia subclusters. (C) The proportion of each subcluster in both young and aged microglia. (D) The proportion of significantly changed subclusters (C3, C4 and C5) in aged microglia compared to young microglia; n = 8 samples per condition. (E) Bar plots show the expression level of nine resting microglia markers in C0 compared to all other microglia subclusters. ***P-value < 0.001, C0 vs. others. (F) GO enrichment analysis was performed on DEGs of C0 vs. all other subclusters. GO terms in the three categories (biological process, cellular component, molecular function) that were significantly overrepresented (P < 0.01) were presented as bubble plots, where the sizes of the bubbles reflect the number of genes under each term. (G) GO Enrichment analysis was performed on DEGs for each subcluster (C1 through C5) vs. C0. Enriched and activated (z-score ≥ 2) GO terms were categorized into functional clusters according to the biologic functions they were involved in. Shown are the functional clusters, which contain at least five terms in microglia subclusters C1 through C5, and each one is denominated according to these functional clusters.

To explore the heterogeneity of microglia in both young and aged mice, unsupervised clustering analysis was performed for all microglia based on Seurat workflow. Six distinct subclusters (C0-C5) of microglia were identified in both young and aged mice (Fig. 2A and Supplementary Fig. 2A) based on the enriched genes for each cluster (Fig. 2B). Three out of these six microglial subsets showed dramatic alterations in cell number (Fig. 2C). In particular, the number of microglia in C3 and in C5 were robustly increased in aged mice (Fig. 2D), while the number of microglia in C4 was significantly decreased in aged mice (Fig. 2D).

We noted that subcluster C0 accounted for almost 50% of the total microglia cell number (Supplementary Fig. 2B), which indicates that this subgroup of microglia may be the dominant type in the brain. All previously reported markers of resting microglia including P2ry13, Fcrls, Ecscr, Rnase4, Slc2a5, P2ry12, Olfml3, and Siglech were highly expressed in C0 microglia compared with the other microglia (Fig. 2E and Supplementary Fig. 2C). In addition, GO enrichment analysis of DEGs between C0 microglia and the other subclusters of microglia showed an extensive inhibition in all the GO functional terms (Fig. 2F and Supplementary Table 2). These data indicate that the C0 subcluster of microglia may be in a resting state, which is consistent with the findings of other previous studies [12, 19]. Furthermore, we compared other subclusters of microglia with resting microglia. Based on their functional features, we defined the cluster C1-C5 subclusters as “surveillant microglia”, “axon-associated microglia”, “HAM”, “synapse-associated microglia”, and “virus-associated microglia”, respectively (Fig. 2G and Supplementary Table 2). Next, we analyzed expression of well-known microglial markers for M1/M2 polarization in all subclusters. Only four out of 12 M1 markers were observed to be significantly upregulated in the C3 subcluster of microglia compared to microglia from young mice (Supplementary Fig. 2D). No M2 markers were found to be highly expressed in all subgroups of aged microglia (Supplementary Fig. 2D). However, multiple age-related markers including Ctsb, Ctsz, Ctsd, Ctsl, Cd9, Apoe, Lpl, Spp1, Cst7, Ank, Axl, Ccl6, and Itgax, were highly expressed in C3 microglia compared with young microglia (Supplementary Fig. 2D).

**Figure 3.**
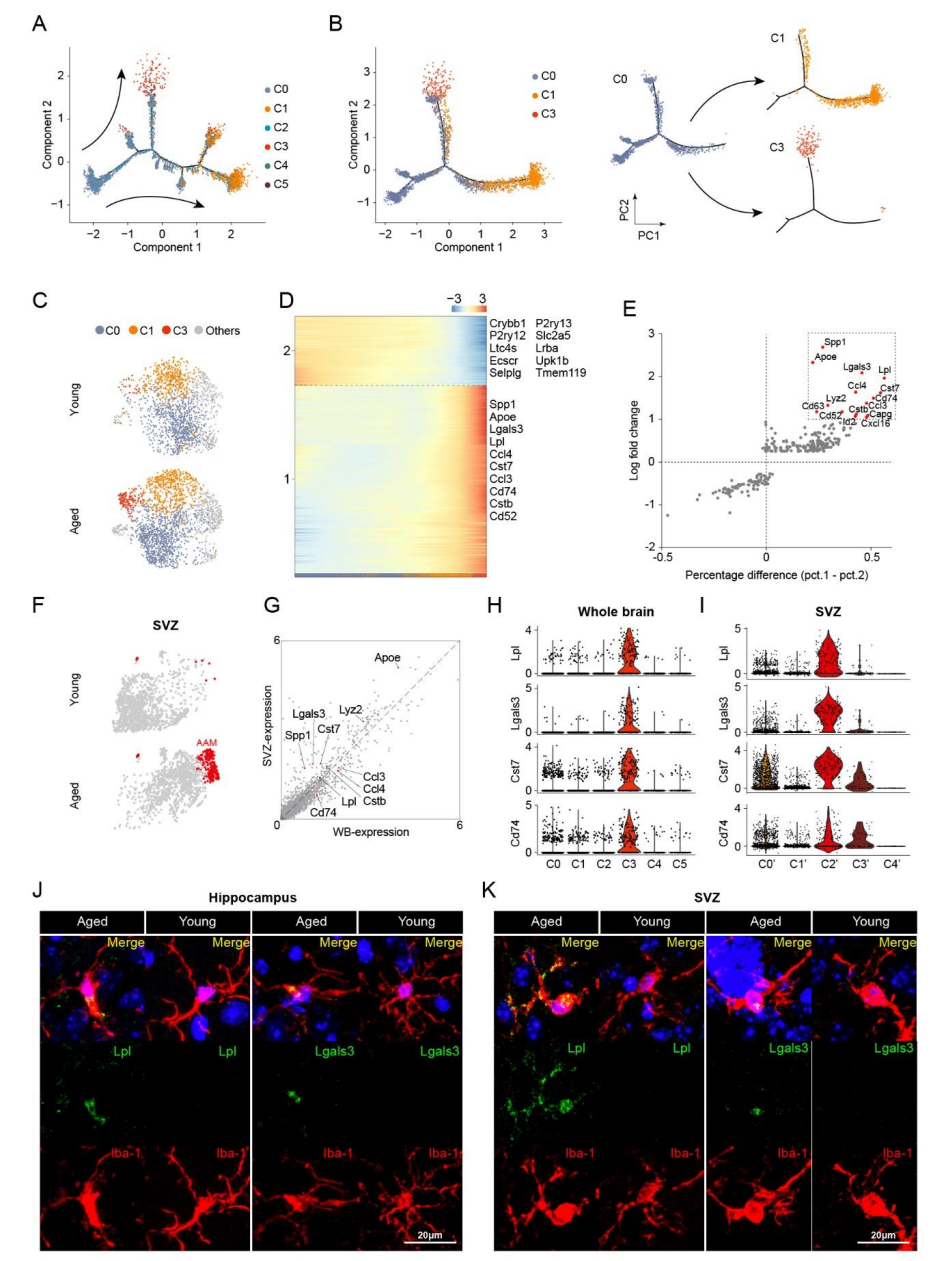
Identification of HAM in both whole brain and SVZ of aged mice. (A) Cellular trajectory of all microglia subclusters (C0 through C5), generated by the Monocle DDRTree dimensionality reduction algorithm. (B) Cellular trajectory of microglia subcluster C0, C1, and C3 (left). Predicted shifting from C0 to C1 and C0 to C3 based on acquired cellular trajectory is shown (right). (C) UMAP showing the microglia subcluster, with C0, C1, and C3 highlighted. (D) Heatmap showing the transcriptomic gradual shift from resting microglia (C0) to HAM C3, with pseudo temporal ordering along the x-axis. (E) Scatter plot showing all DEGs of HAM (C3) vs. resting microglia (C0), with the expression percentage difference (defined as expression percentage in C3 - expression percentage in C0) along the x-axis and log-transformed fold change along the y-axis. Potential marker genes (percentage difference > 0.2 and log fold change > 1) in HAM is noted in red color. (F) t-distributed stochastic neighbor embedding (t-SNE) was used to visualize the result of unsupervised clustering performed on young and aged microglia in the SVZ. Age-associated microglia (AAM) are highlighted in red. (G) A scatter plot shows the correlation in expression level between HAM and AAM. Ten potential markers for HAM are annotated. (H and I) A violin plot shows the expression level of potential marker genes of HAM in whole brain (H) and AAM in SVZ (I). (J and K) Representative confocal microscopic images showing the immunofluorescent signal of Lpl or Lgals3, double-labeled with Iba-1 in the hippocampus (J) and the SVZ (K) of aged mice. Cells were counterstained with DAPI for nuclear labeling. Scale bars: 20 μm.

Interestingly, there was a significant increase in the number of highly-activated and virus-associated microglia in aged mice compared with young mice (Fig. 2D). Together, these findings indicated that these two special types of microglia may be important in the aged brain.

### Identification of HAM in both whole brain and in the SVZ of aged mice

To determine the evolution of the above-mentioned subtypes of microglia, we performed Monocle Pseudo-Time analysis, and revealed that HAM and surveillant microglia were in two distinct directions of the trajectory (Fig. 3A and Supplementary Fig. 3A). HAM and surveillant microglia were extracted and put into the pseudo-time analysis with the resting microglia, and the results showed a gradual transition from resting microglia to surveillant microglia or HAM (Fig. 3B and Supplementary Fig. 3B). Interestingly, the HAM was mainly found in aged brains (Fig. 3C). Hence, we applied FateID to visualize the changes in gene expression among these three subclusters of microglia in aged mice (Fig. 3D). Two distinct modules of gene expression were observed, one of which included a series of downregulated homeostatic microglia markers (including P2ry12, P2ry13, and Tmem119), while the other included a variety of upregulated genes involving in neuroinflammatory response (including Spp1, Apoe, and Lgals3) (Fig. 3D). Further, a sharp variation of gene expression was observed in the transition from the resting microglia to HAM (Supplementary Fig. 3C). These data indicate that HAM possesses a unique activation pattern. Activated microglia generally polarize into a pro-inflammatory M1 state or an anti-inflammatory M2 state. However, only three M1 markers (Cd68, Ccl4 and Tlr2) showed upregulated expression in HAM (Supplementary Fig. 2D), while multiple previously reported age-related markers [5] showed upregulation in HAM (Supplementary Fig. 2D). These results suggest that HAM have a unique, age-related activation pattern, rather than conventional M1/M2 patterns.

To further explore the specific markers of HAM, we analyzed differential gene expression between HAM and the other subclusters of microglia (Fig. 3E). Fifteen candidate markers including Spp1, Lpl, Lgals3, Cst7, Cd74, and Apoe were significantly upregulated in HAM (Fig. 3E). The violin plots showed that Spp1, Capg, Lpl, Lgals3, Cst7, and Cd74 were almost exclusively expressed in HAM (Fig. 3H and Supplementary Fig. 3D). Other potential markers, including Apoe, Ccl3, Ccl4, Lyz2, Cd63, Cd52, Cstb, Id2, and Cxcl16 were highly expressed in HAM, but were also expressed in other subclusters of microglia (Supplementary Fig. 3D). To confirm the stability and universality of these markers, we analyzed another published single-cell transcriptomic dataset from the SVZ (Supplementary Fig. 4). A unique type of age-associated microglia was also identified in the SVZ of aged mice (Fig. 3F), showing a strong positive correlation in gene expression with HAM in the whole brain (Fig. 3G). As expected, candidate markers, including Lpl, Lgals3, Cst7, and Cd74, were highly expressed in the age-associated microglia (Fig. 3I). Immunofluorescence staining was performed to verify the expression of these markers, including Lpl and Lgals3, in the hippocampus and in the SVZ of young and aged mice (Fig. 3J and K). We observed that Lpl and Lgals3 only co-localized with Iba-1 in aged brain (Fig. 3J and K), indicating that Lpl and Lgals3 may be good candidate markers for HAM.

### A remarkable transcriptomic change in HAM involving in immuno-inflammatory responses

**Figure 4.**
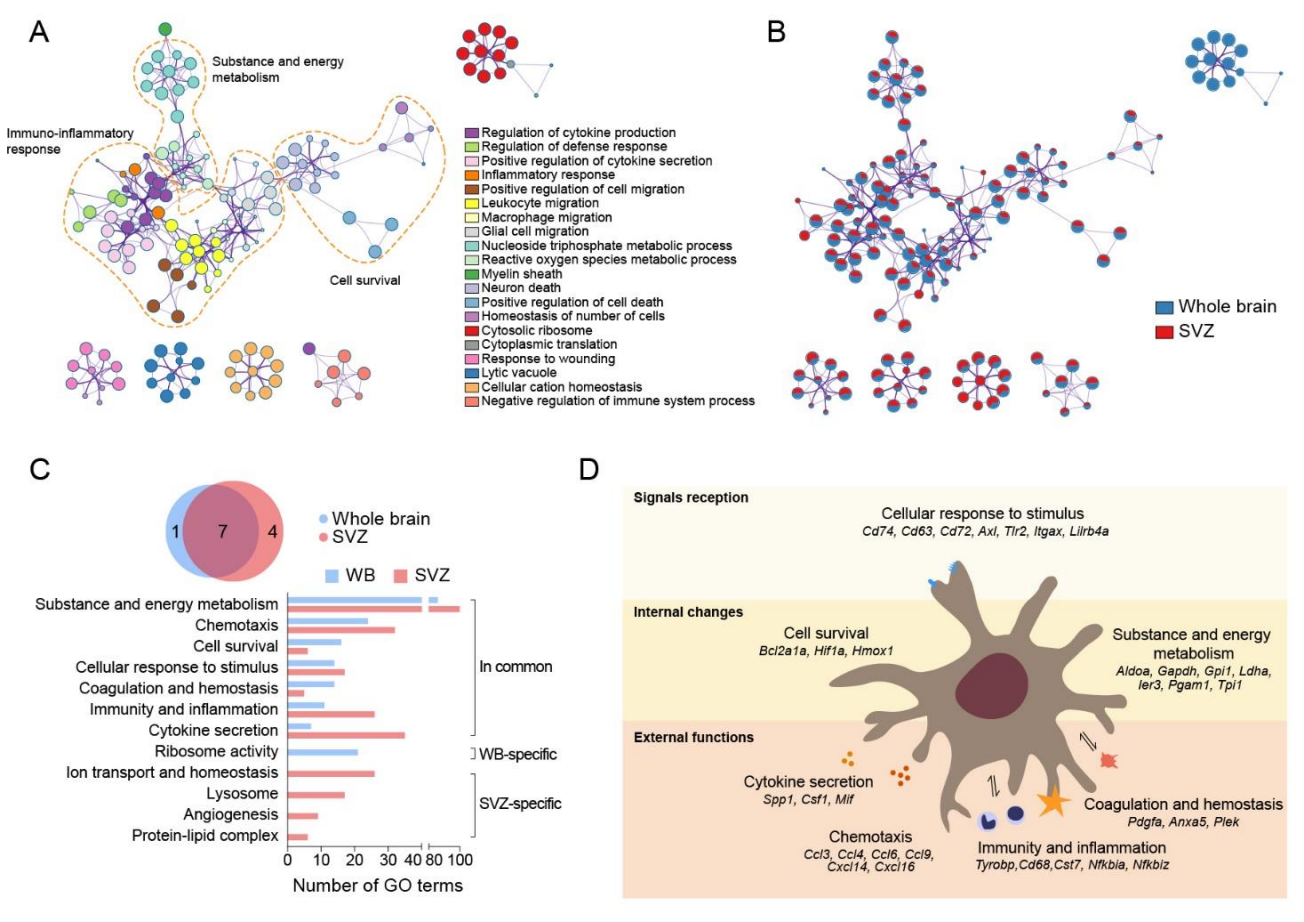
Transcriptomic changes in HAM involving in immuno-inflammatory responses. (A and B) GO enrichment analysis was performed using Metascape on two gene lists from all DEGs of HAM and was compared to resting microglia in both aged whole brain and SVZ. (A) The significantly overrepresented (p < 0.01) GO terms were grouped into color-coded clusters based on their membership similarities and rendered as a network plot. (B) Each node represents an enriched term, and the contribution percentage of different regions is shown in each node. (C) A Venn plot (top) showing the overlap between functional clusters in aged whole brain and in aged SVZ. Bar plot (bottom) showing the number of GO terms in each functional cluster. (D) Shown is the predicted cascade of biological functions in HAM. Biological functions of HAM were divided into three main categories including signal reception, internal changes, and external functions.

To investigate the functional implications of HAM, we performed GO enrichment analysis on two gene lists from all DEGs of HAM compared with resting microglia in aged whole brain and SVZ. The three largest functional clusters were for cell survival, substance and energy metabolism, and immuno-inflammatory response (Fig. 4A). The functional cluster of immuno-inflammatory response consisted of a variety of biological processes such as leukocyte migration, positive regulation of cell migration, regulation of cytokine production, and inflammatory response (Fig. 4A). Enrichment network visualization showed that almost all the major processes such as cell survival, substance and energy metabolism, and immuno-inflammatory response were generally shared between these two lists (Fig. 4B). These data indicated that either HAM from the whole brain or age-associated microglia from the SVZ had altered their transcriptome expression to evoke an immuno-inflammatory response. We categorized all significantly overrepresented GO terms and found that seven functional clusters (substance and energy metabolism, chemotaxis, cell survival, cellular response to stimulus, coagulation and hemostasis, immunity and inflammation, and cytokine secretion) were commonly upregulated in HAM from the whole brain (Supplementary Table 3) and in age-associated microglia from the SVZ (Fig. 4C and Supplementary Table 4). These commonly upregulated functions were reflected in several cellular physiological processes. Firstly, HAM was able to enhance receipt of intra- or extra-cellular signals via upregulated expression of cell-membrane receptors including Cd74, Cd63, Cd72, Axl, Tlr2, Itgax, and Lirb4a (Fig. 4D). Furthermore, two types of internal changes were predicted to occur in HAM. First, HAM viability was predicted to increase via upregulation of pro-survival genes such as Bcl2a1a, Hif1a, and Hmox1 (Fig. 4D). Second, a boost in HAM metabolism of substances and energy was predicted to occur via the upregulation of genes involved in metabolism such as Aldoc, Gapdh, Gpi1, and Ldha (Fig. 4D). Finally, there was upregulated gene expression in HAM of secreted cytokines (Spp1, Csf1, and Mif), chemokines (Ccl3, Ccl4, Ccl6, Ccl9, Cxcl14, and Cxcl16), and those factors predicted to participate in immuno-inflammation (Tyrobp, Cd68, Cst7, Nfkbia, and Nfkbiz) (Fig. 4D). Moreover, HAM also upregulated genes involving in coagulation and hemostasis (Pdgfa, Anxa5, and Plex) (Fig. 4D). Together, there was a remarkable transcriptomic change in HAM, which was mainly involved in the immuno-inflammatory responses.

**Figure 5.**
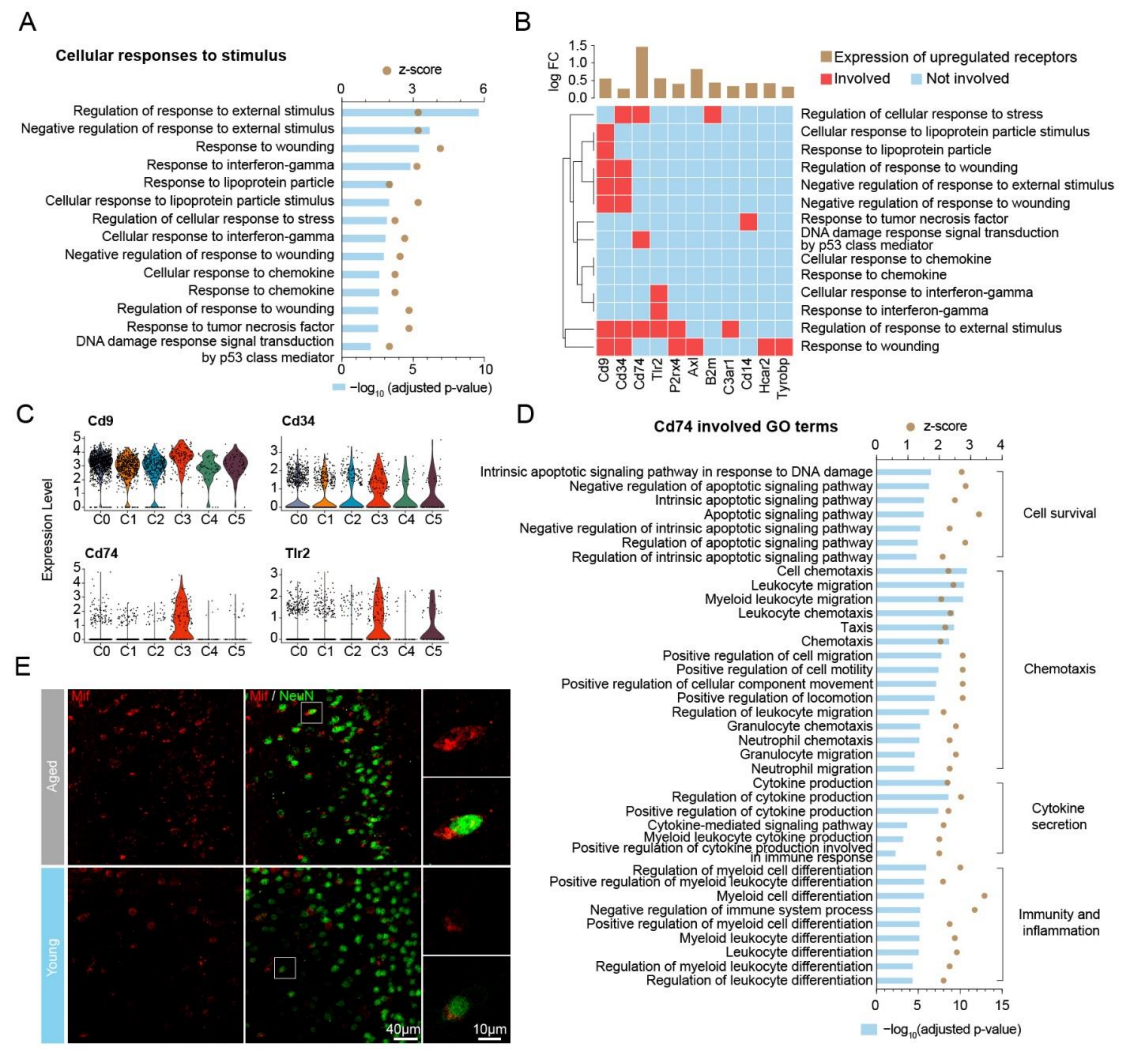
CD74 plays a pivotal in receiving stimuli and triggering cellular activity in HAM. (A) A bar plot showing all 14 activated (z≥2) GO terms involved in the HAM functional cluster for cellular response to stimuli. (B) A heatmap showing the relationship between the 14 GO terms in (A) and the 11 involved receptor genes. The brown bars above show the log-transformed fold change of the involved receptor genes. (C) Violin plots showing the expression level of four receptor genes (Cd9, Cd34, Cd74, Tlr2) involved in at least three GO terms from (B). (D) Bar plot showing all the Cd74-associated GO terms in HAM functional clusters, including cell survival, chemotaxis, cytokine secretion, and immunity and inflammation. (E) Representative confocal microscopic images showing the immunofluorescent signal of Mif double-labeled with NeuN in young and aged brain of mice. Cells were counterstained with DAPI for nuclear labeling. Squares: regions that were zoomed-in. Scale bars: 40 μm for low power; 10 μm for high power.

### CD74 as a signal transverter in HAM: receiving stimuli and triggering cellular activity

To investigate how HAM receive stimuli, we carefully examined all GO terms associated with cellular responses to a stimulus (Fig. 5A). This functional cluster consisted of a variety of biological processes, including regulation of response to an external stimulus, response to wounding, response to interferon-gamma, and more (Fig. 5A). On the other hand, we extracted all receptors that significantly changed in HAM compared with resting microglia in the whole brain (Supplementary Fig. 5A). We then depicted a heatmap to display the relationship between all of the upregulated receptor genes (Cd74, Cd63, Lilrb4a, Cd72, Axl, Itgax, Cd83, Tlr2, Cd9, Lgals3bp, Ccrl2, B2m, Cd14, Hcar2, P2rx4, Csf2ra, Tspo, C3ar1, Tyrobp, Crlf2, and Cd34) and GO functional terms involving in cellular responses to a stimulus (Fig. 5B). The results showed that 11 receptor genes were involved in at least one of the GO functional terms regarding cellular responses to a stimulus (Fig. 5B). Among these 11 receptor genes, only Cd9, Cd34, Cd74, and Tlr2 were involved in three or more GO functional terms (Fig. 5B), indicating that these four receptors may have critical roles in receiving stimuli. In addition, violin plots showed that Cd74, Axl, and Hcar2 were mainly expressed in HAM rather than the other types of microglia (Fig. 5C and Supplementary Fig. 5C). Therefore, Cd74 was selected as a potential candidate receptor for receiving stimuli in HAM. We then confirmed that Cd74 was also mainly expressed in the age-associated microglia of the SVZ (Supplementary Fig. 5B).

**Figure 6.**
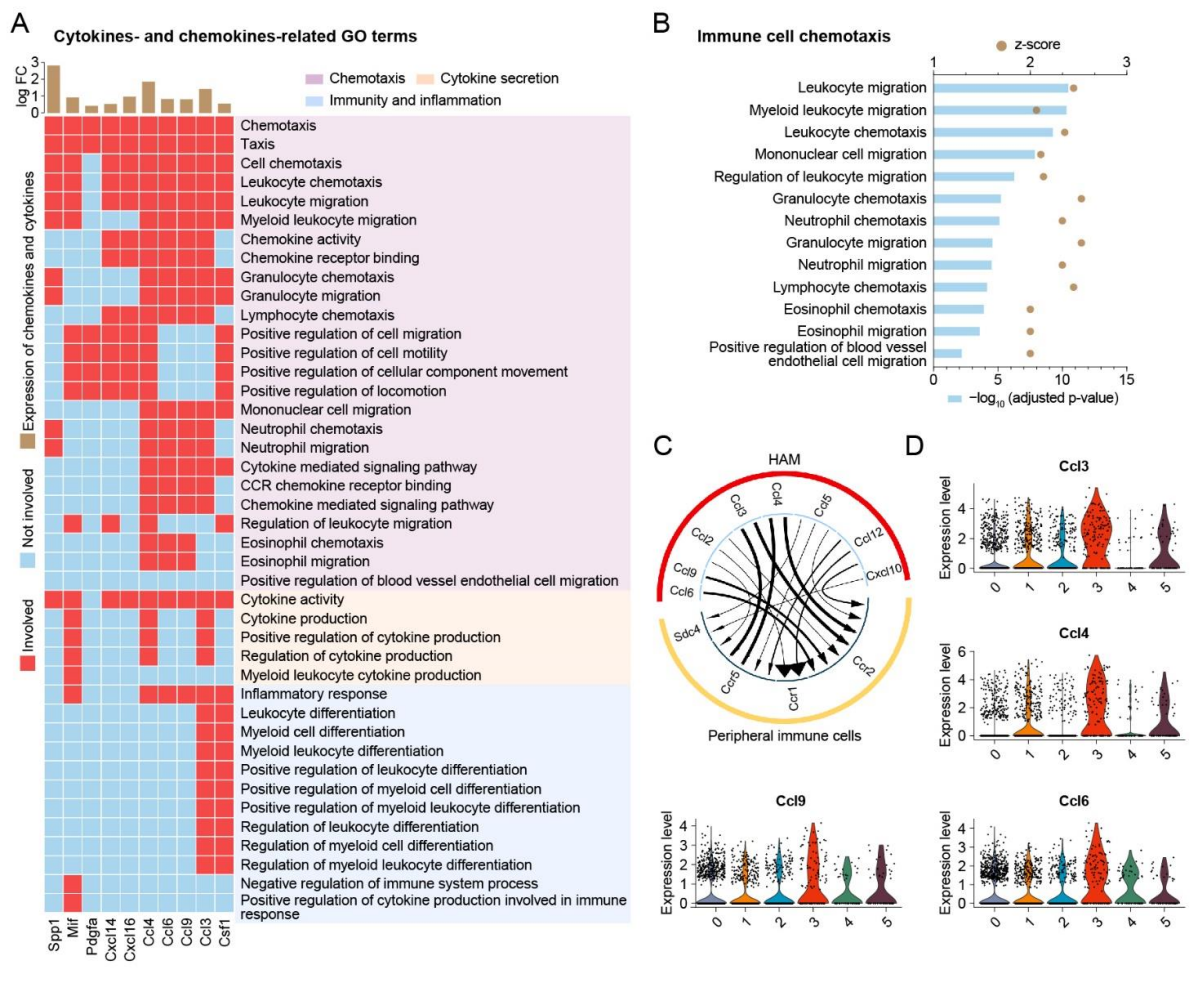
HAM participates in immuno-inflammation by releasing chemokines to recruit peripheral immune cells. (A) The brown bars above show the log-transformed fold change of all chemokine and cytokine genes in the DEGs of HAM compared to resting microglia. A heatmap shows the relationship between GO terms in immuno-inflammatory functional clusters of HAM with chemokine and cytokine genes. (B) Bar plot showing the GO terms involved in the recruitment of peripheral immune cells. (C) Chord plot based on R package iTALK depicting the predicted interaction between HAM and peripheral immune cells (including monocytes, neutrophils, and dendritic cells) in aged whole mouse brains. (D) Violin plots showing the expression level of four chemokine genes (Ccl3, Ccl4, Ccl6, Ccl9) in aged whole mouse brain.

To confirm whether Cd74 evokes downstream biological processes, we monitored all GO terms related to cell survival, substance and energy metabolism, coagulation and hemostasis, cytokine secretion, chemotaxis, and immunity and inflammation (Supplementary Fig. 6). The results showed that Cd74 was involved in eight out of 16 GO functional terms regarding cell survival (Supplementary Fig. 6A), six out of seven GO functional terms regarding cytokine secretion (Supplementary Fig. 6D), 15 out of 24 GO functional terms regarding chemotaxis (Supplementary Fig. 6E), and 10 out of 11 GO functional terms regarding immunity and inflammation (Supplementary Fig. 6F). We then listed all GO terms in the above-mentioned functional clusters including cell survival, chemotaxis, cytokine secretion, and immunity and inflammation (Fig. 5D). Together, these data indicated that Cd74 might be a signal transverter in HAM that functions to receive stimuli and trigger downstream cellular activity. Previous studies have reported that Cd74 is a cell-surface receptor for cytokine macrophage migration inhibitory factor (Mif). We performed immunofluorescence staining of Mif in brains of young and aged mice and found that Mif was specifically expressed in neurons of aged mice (Fig. 5E). These data indicated that Mif released from aged neurons acts through the HAM Cd74 receptor to promote immunochemotactic activity of microglia.

### HAM participate in immuno-inflammation by releasing chemokines to recruit peripheral immune cells

To further explore how HAM participate in immuno-inflammation, we extracted all cytokines and chemokines that were upregulated in HAM compared with the resting microglia in the aged whole brain. Six chemokine genes (Ccl3, Ccl4, Ccl6, Ccl9, Cxcl14, Cxcl16) and four cytokine genes (Csf1, Mif, Pdgfa, Spp1) were significantly upregulated in HAM (Fig. 6A). These ten HAM-released factors were primarily involved in the biological function of chemotaxis including 25 GO functional terms such as chemotaxis, taxis, cell chemotaxis, leukocyte chemotaxis, leukocyte migration, etc. (Fig. 6A). These data reveal that the HAM may release chemokines to promote immuno-inflammation.

In order to further investigate which cell types would be influenced by HAM-released chemokines and cytokines, we extracted all GO functional terms regarding immune cell chemotaxis. Interestingly, peripheral immune cells including leukocytes, granulocytes, mononuclear cells, lymphocytes, eosinophils, and neutrophils were shown to be recruited by HAM (Fig. 6B). These results are consistent with the recently reported findings that peripheral immune cells such as macrophages, monocytes, dendritic cells, and neutrophils can be detected in aged mouse brain [6, 18]. Subsequently, we used the dataset of the whole brain and applied the iTALK toolkit to explore cell-cell interactions between HAM and peripheral immune cells (Fig. 6C). We found several ligand-receptor pairs, including Ccl3-Ccr5, Ccl3-Ccr2, Ccl4-Ccr5, Ccl4-Ccr2, Ccl6-Ccr2, and Ccl9-Ccr2 that were involved in recruiting peripheral immune cells (Fig. 6C). The violin plots showed that Ccl3, Ccl4, Ccl6, and Ccl9 were highly expressed in HAM compared with other subclusters of microglia in the aged whole brain (Fig. 6D). Together, these data indicate that HAM release several chemokines, such as Ccl3, Ccl4, Ccl6, and Ccl9 to recruit peripheral immune cells into the aged brain, evoking immuno-inflammation.

Curiosity surrounding the impact of HAM on the other subclusters of microglia drove us to investigate cell-cell interaction among these subtypes of microglia in the aged whole brain (Supplementary Fig. 7). Surprisingly, HAM was shown to markedly impact other subclusters of microglia through the release of factors, including C1qb, Apoe, Selplg, and Psap (Supplementary Fig. 7A). We further observed that Apoe-Lrp1, Ccl3-Ccr5, and Ccl4-Ccr5 were the main ligand-receptor pairs participating in the interaction between HAM and other subclusters of microglia (Supplementary Fig. 7B). The Apoe, Lpl, Ccl3, and Ccl4 ligands were mainly expressed in HAM rather than in other subclusters of microglia (Supplementary Fig. 7C), while Lrp1 and Ccr5 were found to be expressed in all types of microglia (Supplementary Fig. 7D).

## DISCUSSION

The present study provides single-cell transcriptional profiling of a unique type of highly-activated microglia from the brain of aged mice under homeostatic conditions. Major findings of our study included that (i) a unique type of microglia (HAM) identified by Lpl and Lgals3, were observed to reprogram their transcriptome to evoke inflammation in aged mice; (ii) no brain regional variation was detected in HAM in whole brain or in the SVZ; (iii) the Mif/Cd74 signaling axis triggers release of chemokines from HAM to recruit infiltration of peripheral immune cells into the aged brain.

Transcriptomic analysis of microglia in aged and young mice identified 56 DEGs, representing a moderate alteration of the genome (4.2% of the total 1,333 detected genes). These DEGs were primarily involved in immune inflammatory responses, including adaptive immune response, leukocyte mediated immunity, and T cell mediated immunity. Iba-1 staining also showed that aged microglia were presented as an active phenotype with larger cell bodies compared to young microglia in all regions of the brain, including cortex, striatum, SVZ, and hippocampus. Our previous study reported similar findings in that microglia from aged mice were found to be in a heightened chronic inflammatory state [10]. Importantly, this age-related microglial phenotype was also observed in the aged human brain and it involved in the pathological processes associated with brain aging [9]. However, in aged mice, not all microglia transformed their phenotype into this specially activated state. Our data revealed that only a small fraction of microglia (6.35% of total microglia) showed marked transcriptome upregulation that would participate in immuno-inflammatory responses. This unique type of microglia was also detected by scRNA-seq in brains of transgenic mice with Alzheimer’s disease (AD), in which case they contributed to ~7% of total microglia [5]. Several important genes, including Spp1, Lpl, Apoe, and Cst7, were upregulated in HAM and in AD-associated microglia compared with the resting microglia [5]. Dunja and his colleagues also observed this unique type of age-associated microglia (~12% of total microglia) at the protein level by applying high-dimensional single-cell mass and fluorescence cytometry [6]. We further analyzed the functional clusters of this unique type of microglia, revealing that these cells can evoke chemokine-mediated inflammatory responses. A mass of GO functional terms regarding cytokine production and chemotaxis were highly upregulated in HAM, including cell chemotaxis, leukocyte migration, chemokine activity, and chemokine-mediated signaling pathway. These findings were strikingly consistent with the results from another group, which reported that a special subcluster of chemokine-enriched inflammatory microglia (~5% of total microglia) persisted throughout the lifespan and increased in the aged brain [12]. All these findings from our present study from other groups have indicated that small populations of inflammatory microglia emerge in the aged brain.

A tremendous number of questions still need to be addressed before this unique type of microglia in the aged brain can be comprehensively unmasked. First of all, the underlying mechanism of the senescence-associated inflammation remains elusive. Although emerging evidence supports that AD-associated microglia might be activated by surrounding Aβ particles, no previous studies have thoroughly investigated the mechanism behind activation of this unique type of aged microglia in the aged brain. Our present data reveal that the activation of the Mif/Cd74 signaling axis may be involved in HAM-induced brain inflammation. Our present analysis shows that the cell surface receptor, Cd74, is in charge of receiving both intracellular and extracellular signals before triggering downstream cellular activity. Previous studies have reported that Cd74 was highly expressed in M1 (inflammatory type) microglia in the hippocampal CA1 region following ischemic stroke with induced neuronal damage [20]. In addition, a higher amount of CD74-positive microglia were detected in the glioblastoma and were associated with beneficial patient survival [21]. A previous report of Cd74 as a positive prognostic marker was likely due to its association with an M1-polarized immune milieu in glioblastoma [20]. Together, these previous reports support our findings that Cd74 may function as a signal transducer in HAM, in charge of receiving stimuli to evoke brain inflammation. Mif binds to Cd74 and is an important regulator of innate and adaptive immunity [22]. Our results showed that neuron-released Mif was detected only in the brains of aged mice. Thus, we believe that aging increases neuronal secretion of Mif which binds to Cd74 and activates HAM to evoke brain inflammation. Secondly, our study revealed no noteworthy observations to suggest that there is region-dependent diversity of this unique type of microglia. Our data show similar transcriptomic changes in HAM in whole brain and in the SVZ of the aged mice. A previous study reported that aging of microglia occurs in a region-dependent manner [23]. They found that a large group of genes was upregulated with age in microglia across all brain regions, while another cluster of genes increased with age only in the cerebellum [23]. In addition, this study also reported that hippocampal microglia showed a declining sensitivity with age [23]. However, those findings were detected by bulk RNA sequencing, which is not able to distinguish subgroups of microglia. We found that the proportion of HAM in microglia was 6.35% in the whole brain and 17.86% in the SVZ. Therefore, varying prominence of the HAM in different brain regions may explain the previous findings from bulk RNA sequencing that aging of microglia occurrs in a region-dependent manner. Lastly, HAM constitute just 6-15% of total microglia in the aged brain, and the question remains as to how this small fraction of microglia evoked brain inflammation. Our data revealed that this small fraction of aged microglia functions to trigger a cascade of amplification by activating other types of microglia and recruiting peripheral immune cells to infiltrate into the aged brain.

Several limitations of the present study should be noted. First, our findings were mainly based on the analysis of scRNA-seq from two published databases. Transcriptomic changes do not always determine the molecular alterations at the protein level and/or functional level. In the present study, we applied immunofluorescent staining to detect the changes in morphological features of microglia in aged versus young mice, and then verified several differentiallly expressed genes, including Lpl, Lgals3, and Mif at the protein level in HAM. Nevertheless, future studies need to be designed to examine the functional significance of the transcriptional alterations in this report. Second, the current study focused only on the HAM. Two subclusters of microglia were also significantly changed in the aged brain compared with the young brain. Virus-associated microglia were significantly increased in number with age, while synapse-associated microglia showed a remarkable decline in the aged mouse brain. These two subclusters of microglia require further studies. Third, all experiments in our study were performed in male animals because both scRNA-seq datasets were from male mouse brains. This might result in a failure to identify a potential sex bias in microglial phenotype and functions. We reviewed all published papers about sex differences in microglia as detected by transcriptomics [12, 24-25] and found that observations were not consistent among these studies. Guneykaya et al. compared males and females and found 1,109 genes differentially expressed in the hippocampus and 55 genes differentially expressed exclusively in the cortex; only 46 genes were differentially expressed in both regions between males and females [24]. Furthermore, Villa et al. also performed bulk RNAseq for young adult male and female mice, which found that male microglia up-regulated inflammatory processes including regulation of cell migration and cytokine production [25]. Female microglia were grouped in ontogenies associated with morphogenesis, development, or cytoskeleton organization, instead of inflammation [25]. However, Hammond et al. reported almost no difference in the clustering between the male and female samples, when compared with microglia from male and female mice at three major developmental ages: E14.5, P4/P5, and P100 using scRNAseq technique [12]. Unfortunately, there are no available single-cell RNA-seq datasets from geriatric female mouse brains. The sex differences of young and aged microglia will be further elucidated in future studies. Overall, our study unmasks transcriptome alterations in HAM, and provides a source to screen for potential targets to modulate microglial functions in aged subjects. However, to draw solid conclusions, additional mechanism-oriented studies are needed.

In conclusion, this study applied transcriptomic analysis of aged and young microglia in mice. A unique type of HAM that is identified by Lpl and Lgals3 showed marked upregulation of many genes involving in cell survival, energy metabolism, and immuno-inflammatory responses. This HAM is activated by neuron-released Mif through its Cd74 receptor, after wich peripheral immune cells are recruited to evoke immuno-inflammation. These findings may reveal new targets for reducing age-related brain inflammation to maintain brain health.

## Supplementary Materials

The Supplementary data can be found online at: www.aginganddisease.org/EN/10.14336/AD.2021.0520.


