# Supplementary material for: A Unique Type of Highly-Activated Microglia Evoking Brain Inflammation via Mif/Cd74 Signaling Axis in Aged Mice
Source: Aging Dis. 2021 Dec 1;12(8):2125–39. doi: 10.14336/AD.2021.0520 (PMC8612608; doi:10.14336/AD.2021.0520)
Supplement: Supplementary file 1 [file AD-12-8-2125-s.pdf]

Supplementary Data

A Unique Type of Highly-Activated Microglia Evoking Brain Inflammation via Mif/Cd74 Signaling Axis in Aged Mice

Chenghao Jin[1,#], Yijie Shao[1,#], Xiaotao Zhang[1], Jiani Xiang[1], Ruize Zhang[1], Zeyu Sun[1], Shuhao Mei[1], Jingyi Zhou[1,*], Jianmin Zhang[1,2,3,*], Ligen Shi[1,*]

© 2021. Jin C et al. Published online at http://www.aginganddisease.org/EN/10.14336/AD.2021.0520

Supplementary Data

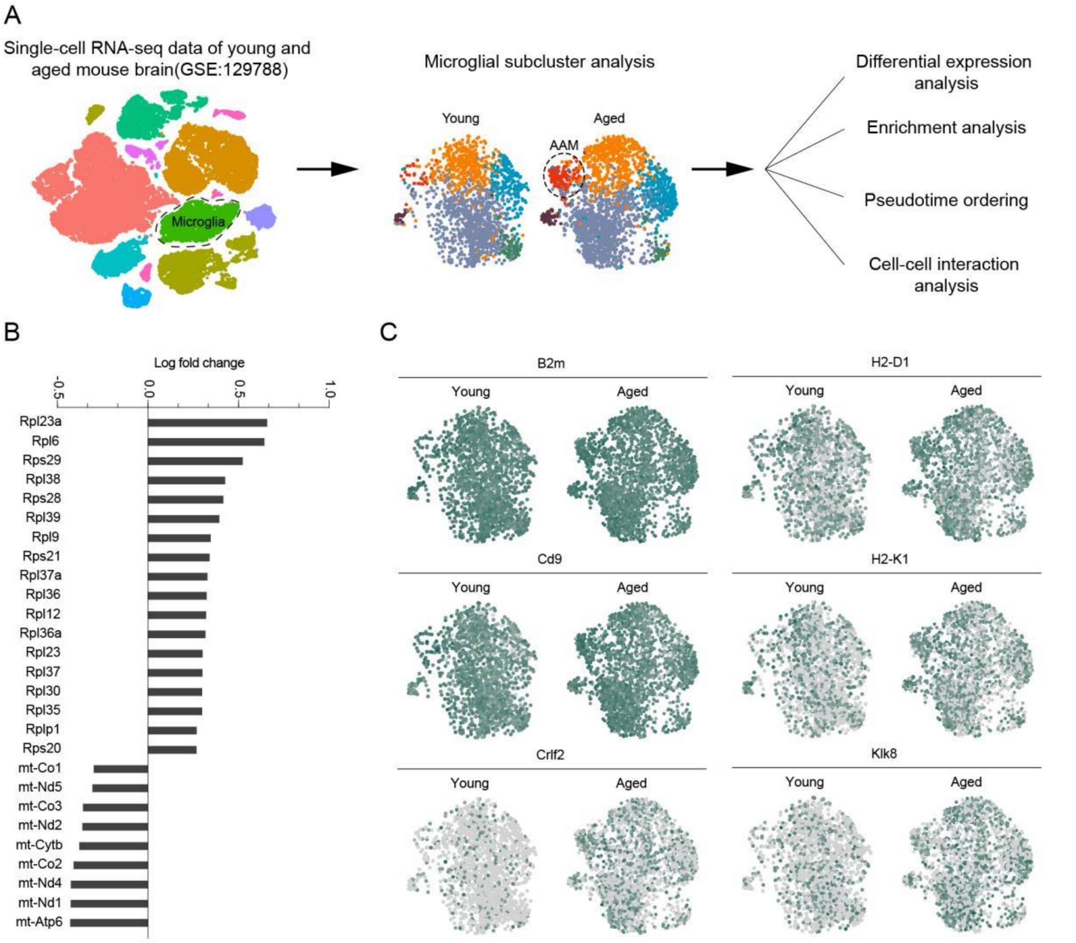

Supplementary Figure 1. Differences in microglia between young and aged mice. (A) Workflow of this study. Firstly, the microglia cluster was extracted from scRNA-seq data using clustering analysis based on R package Seurat. Then subcluster analysis on microglia cluster identified a unique group of highly-activated microglia (HAM) in aged mice. Finally, various bioinformatic methods were applied for exploring all aspects of HAM. **(B)** Bar plot showing the natural log fold change of ribosomal and mitochondrial genes in DEGs of young microglia versus aged microglia. **(C)** UMAP showing the expression of B2m, Cd9, Crlf2, H2-D1, H2-K1, Klk8 in both young and aged microglia.

© 2021. Jin C et al. Published online at http://www.aginganddisease.org/EN/10.14336/AD.2021.0520

Supplementary Data

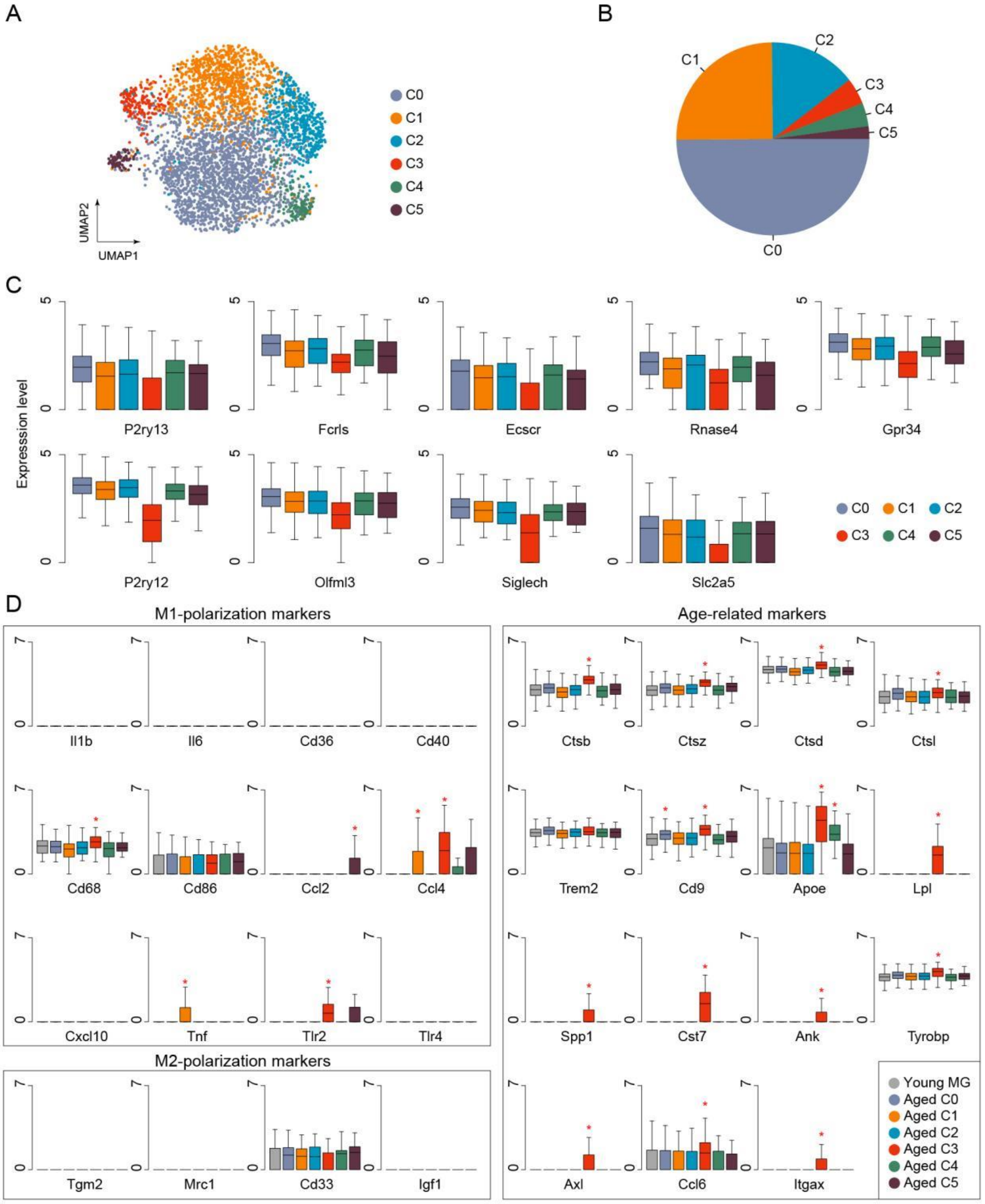

Supplementary Figure 2. Features of microglial subclusters. (A) Uniform manifold approximation and projection (UMAP) of all six subcluster(C0-C5) in all microglia. **(B)** Pie chart showing the proportion of each microglial subcluster in (a). **(C)** Bar plot showing the differential expression level of nine previously reported marker genes for resting microglia in each microglial subcluster. **(**D) Bar plot showing the expression level of detected microglial M1-polarization markers, M2-polarization markers and age-related markers in each microglial subcluster. Asterisk indicated the expression level was significantly higher (log fold change > 0.25 and Bonferroni adjusted p-value < 0.05) in the cells of this subcluster than all the other cells.

© 2021. Jin C et al. Published online at http://www.aginganddisease.org/EN/10.14336/AD.2021.0520

Supplementary Data

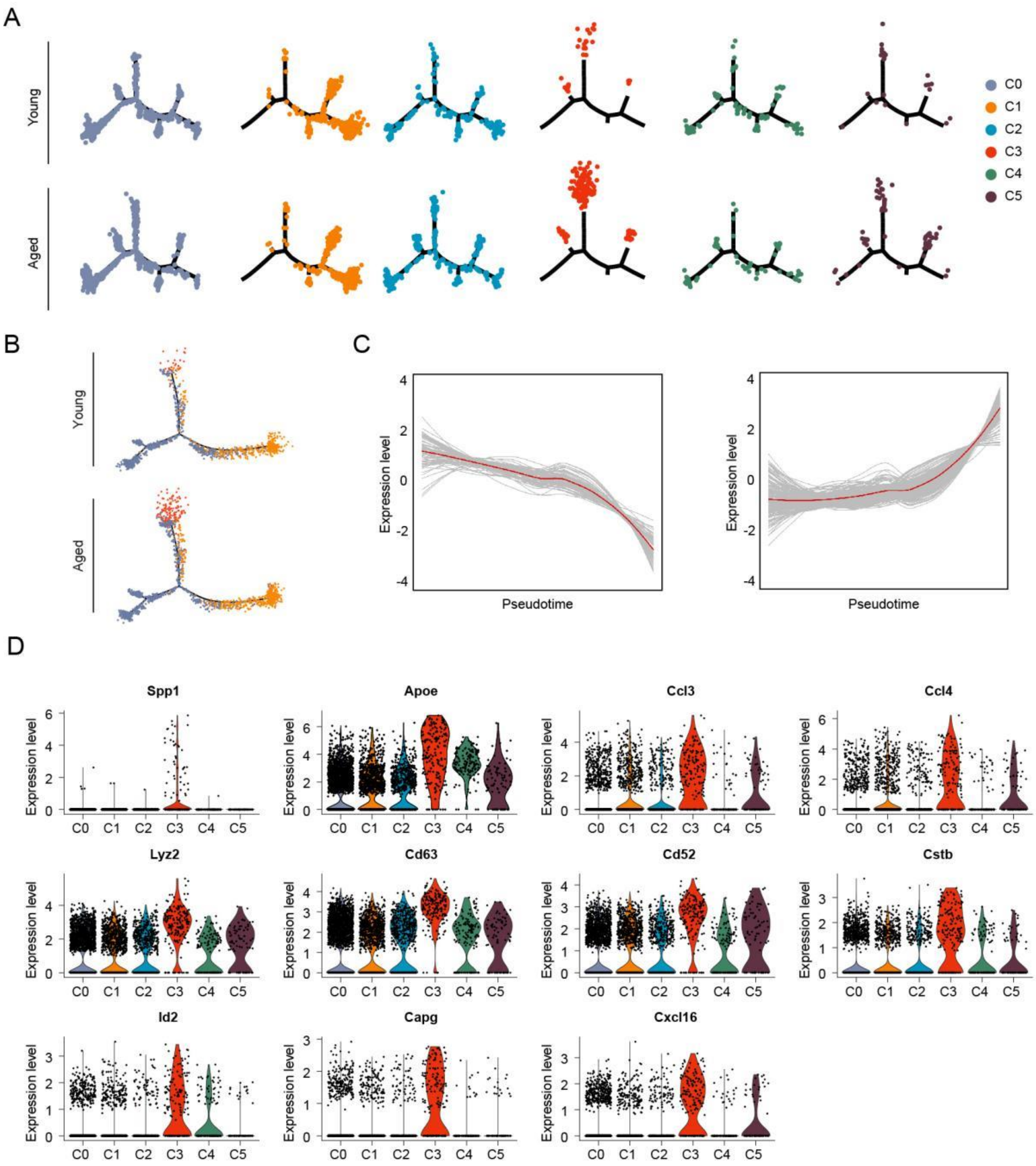

Supplementary Figure 3. Features of aging-associated microglia. (A) Cellular pseudotime trajectories of each subclusters both in young and aged microglia generated by DDRTree dimensionality reduction algorithm of Monocle R package. **(B)** Cellular pseudotime trajectories of subcluster C0, C1 and C3 both in young and aged microglia. **(C)** Genes in Fig.3d were divided into two groups according to their similarity in variation tendency, plots showing the variation pattern of each group. **(D)** Violin plot showing the expression level of 11 highly potential marker genes of HAM.

Supplementary Data

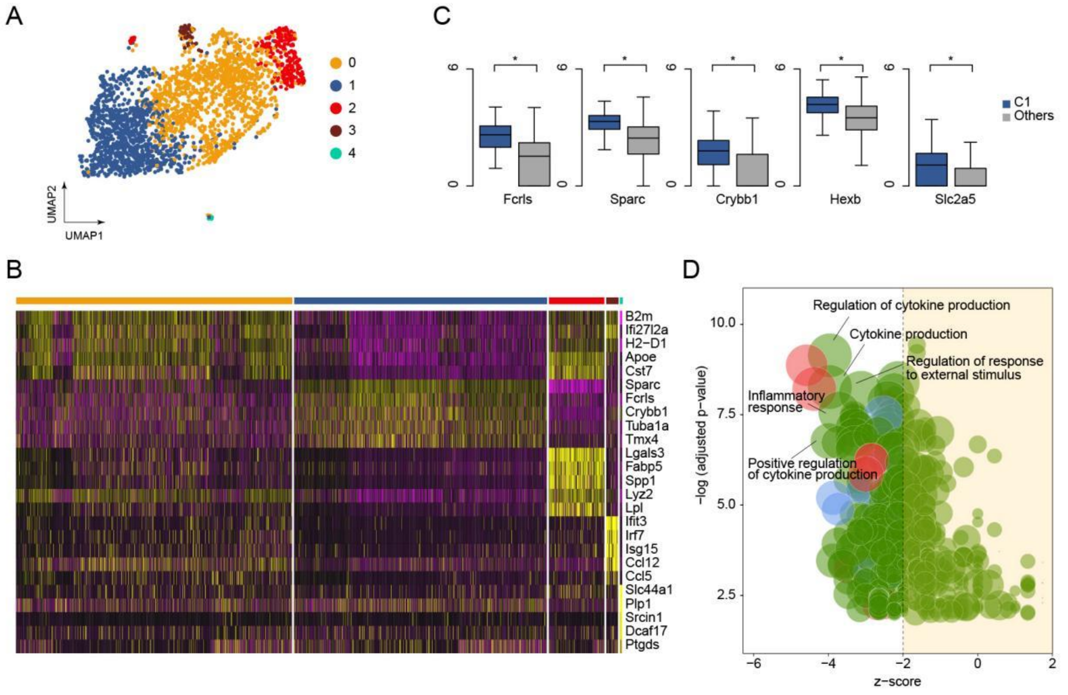

Supplementary Figure 4. Analysis of scRNA-seq data of microglia in SVZ. (A) UMAP of all five subcluster(C0-C4) in both young and aged microglia. **(B)** Heatmap visualizing the expression level of top five marker genes in each microglial subclusters. **(C)** Bar plot showing the differential expression level of previously reported marker genes for resting microglia in C1 compared with that in all the other microglial subclusters (*Bonferroni adjusted P value<0.05). **(D)** Bubble plot showing the enriched GO terms after enrichment analysis using DEGs of microglia in C1 versus all the other microglia.

© 2021. Jin C et al. Published online at http://www.aginganddisease.org/EN/10.14336/AD.2021.0520

Supplementary Data

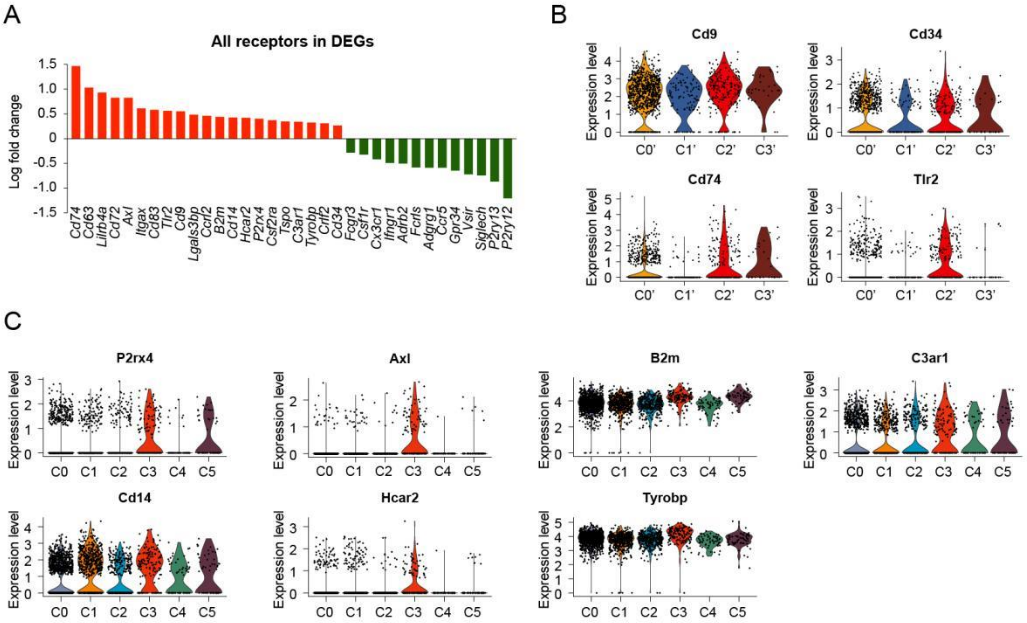

Supplementary Figure 5. Expression of receptors. (A) Bar plot showing the fold change of all receptors in DEGs of aged microglia compared with young microglia. **(B)** Violin plot showing the expression levels of Cd9, Cd34, Cd74, Tlr2 in microglial subclusters of SVZ. **(C)** Violin plot showing the expression levels of all receptors in microglial subclusters of whole brain.

© 2021. Jin C et al. Published online at http://www.aginganddisease.org/EN/10.14336/AD.2021.0520

Supplementary Data

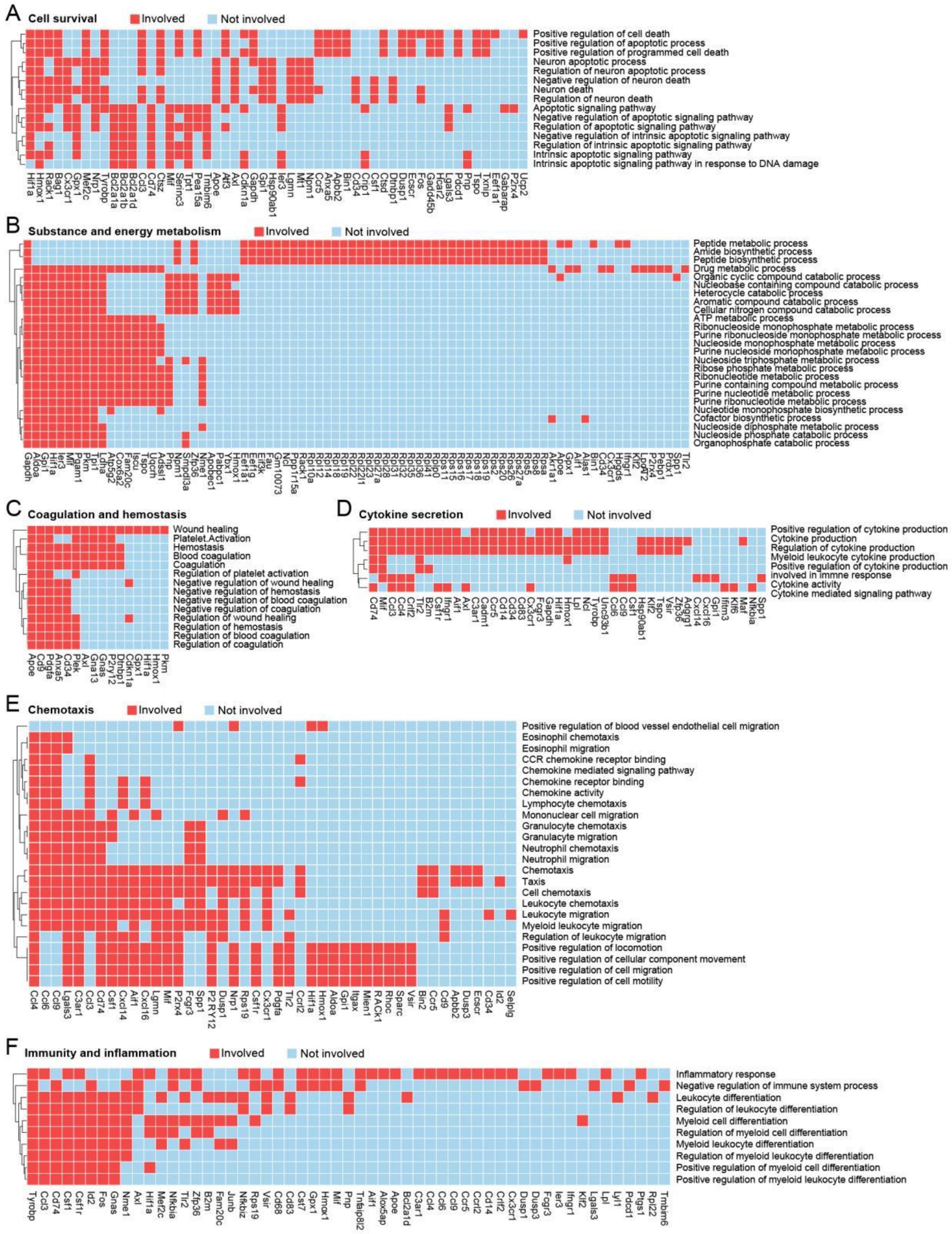

Supplementary Figure 6. Gene involvement in GO terms. (A-F) Heatmap showing the relationship between GO terms associated with cell survival (A), substance and energy metabolism (B), coagulation and hemostasis (C), cytokine secretion (D), chemotaxis (E) and involved genes immunity and inflammation (F).

© 2021. Jin C et al. Published online at http://www.aginganddisease.org/EN/10.14336/AD.2021.0520

Supplementary Data

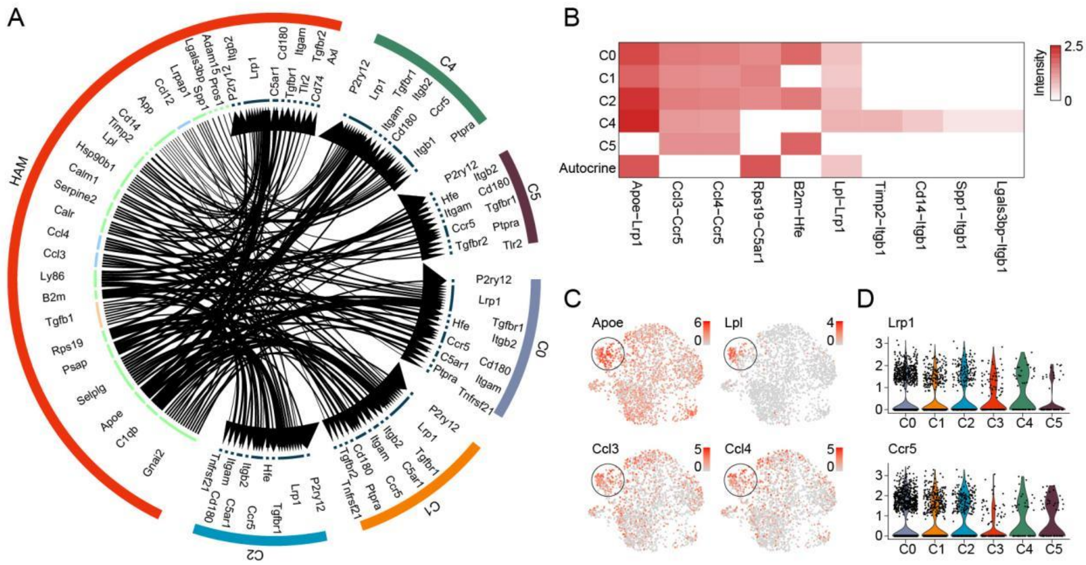

Supplementary Figure 7. Interaction between HAM and other microglial subclusters. (A) Chord plot based on R package iTALK depicting the predicted interaction between HAM and all the other microglial subclusters in aged whole brain. **(B)** Heatmap showing the predicted intensity of interaction of receptor-ligand pairs between HAM and all the other microglial subclusters in aged whole brain. **(C)** UMAP showing the selective expression of four representative ligands of HAM (Apoe, Lpl, Ccl3, Ccl4) in (B). **(D)** Violin plots showing the even expression of two representative receptors of HAM (Lrp1, Ccr5) in (B)

© 2021. Jin C et al. Published online at http://www.aginganddisease.org/EN/10.14336/AD.2021.0520

SUPPLEMENTARY DATA

Supplementary Tables

Supplementary Table 1. Differentially expressed genes in microglia from aged mice versus young mice.

Genes	P-value	Average logFC	Percentage in aged	Percentage in young	Adjusted P-value
Crlf2	8.22E-86	0.695793543	0.476	0.165	1.05E-81
Rpl23a	6.56E-66	0.658431445	0.43	0.176	8.42E-62
Rpl6	4.04E-83	0.6426102	0.711	0.419	5.19E-79
Lyz2	1.69E-58	0.597357896	0.712	0.49	2.16E-54
Rps29	9.31E-64	0.52379043	0.964	0.911	1.19E-59
Ppfia4	6.62E-71	0.513711859	0.55	0.238	8.49E-67
Ccl4	2.28E-15	0.446561388	0.265	0.156	2.92E-11
Ccl3	1.00E-23	0.430851659	0.332	0.185	1.28E-19
Rpl38	1.41E-41	0.425835654	0.815	0.66	1.80E-37
Cd52	8.61E-30	0.419667466	0.617	0.439	1.10E-25
Rps28	8.92E-29	0.415904396	0.664	0.501	1.14E-24
Apoe	1.48E-06	0.412138576	0.704	0.749	0.018973119
Rpl39	7.50E-66	0.393824187	0.96	0.865	9.62E-62
Lsp1	4.64E-28	0.391642635	0.3	0.146	5.95E-24
Klk8	9.32E-35	0.366723018	0.512	0.304	1.20E-30
Cd63	3.50E-33	0.363524713	0.797	0.662	4.49E-29
H2-D1	8.17E-32	0.356693999	0.799	0.662	1.05E-27
Rpl9	1.23E-23	0.346848615	0.381	0.228	1.57E-19
Rps21	3.33E-41	0.339882732	0.866	0.714	4.27E-37
Rpl37a	7.43E-50	0.32842553	0.973	0.914	9.53E-46
Rpl36	8.74E-32	0.32488124	0.848	0.73	1.12E-27
Rpl12	8.34E-36	0.320968576	0.813	0.688	1.07E-31
Rpl36a	1.16E-34	0.31709392	0.854	0.725	1.49E-30
Tpt1	1.92E-42	0.304401634	0.927	0.851	2.46E-38
H2-K1	1.78E-24	0.304005424	0.61	0.436	2.28E-20
Rpl23	3.77E-45	0.301519819	0.95	0.896	4.83E-41
Rpl37	7.81E-35	0.300871489	0.96	0.889	1.00E-30
Rpl30	2.16E-44	0.299372655	0.914	0.81	2.77E-40
Rpl35	3.21E-20	0.299063477	0.652	0.5	4.12E-16
Upk1b	6.75E-23	0.288091121	0.319	0.169	8.66E-19
Rplp1	5.66E-50	0.268854684	0.98	0.934	7.26E-46
Rps20	3.17E-34	0.268213256	0.903	0.807	4.07E-30
Actb	5.51E-25	0.260931102	0.992	0.982	7.07E-21
B2m	1.41E-42	0.258407813	0.995	0.994	1.81E-38
Cd9	9.09E-31	0.250669479	0.962	0.939	1.17E-26
Fcrls	6.01E-26	-0.254372786	0.899	0.939	7.71E-22
Pld1	1.65E-10	-0.268801177	0.233	0.308	2.11E-06
mt-Co1	3.85E-47	-0.29908548	0.999	0.998	4.93E-43
mt-Nd5	2.22E-13	-0.306373545	0.434	0.51	2.84E-09
P2ry12	4.53E-64	-0.312783131	0.968	0.99	5.81E-60
Mt3	2.11E-09	-0.319009042	0.239	0.31	2.71E-05
Serinc3	1.87E-22	-0.323520542	0.736	0.788	2.40E-18
Csf1r	1.24E-89	-0.347559437	0.954	0.988	1.60E-85
Rsrp1	6.33E-33	-0.354383071	0.713	0.78	8.11E-29
Ndrg2	3.12E-13	-0.354546998	0.205	0.291	4.00E-09
mt-Co3	1.56E-79	-0.358505313	1	0.999	2.01E-75
mt-Nd2	5.25E-39	-0.36215292	0.878	0.921	6.73E-35

© 2021. Jin C et al. Published online at http://www.aginganddisease.org/EN/10.14336/AD.2021.0520

Sepw1	3.30E-16	-0.374408474	0.369	0.45	4.23E-12
mt-Cytb	6.73E-82	-0.378390457	0.997	0.999	8.64E-78
Cpe	8.80E-14	-0.397851753	0.234	0.321	1.13E-09
mt-Co2	8.50E-76	-0.410546435	0.988	0.988	1.09E-71
Pcsk1n	7.47E-20	-0.410632381	0.227	0.337	9.59E-16
Aldoc	3.84E-17	-0.410915633	0.241	0.342	4.92E-13
mt-Nd4	2.03E-87	-0.425814425	0.986	0.995	2.61E-83
mt-Nd1	6.21E-109	-0.426912291	0.996	0.998	7.96E-105
mt-Atp6	2.36E-108	-0.42883186	1	0.999	3.03E-104

Supplementary Table 2. Functional enrichment results of DEGs for six subclusters of microglia.

C0vsOthers	
Terms	Z-score
transcription factor AP-1 complex	-2
response to cAMP	-2
cellular response to calcium ion	-2
response to organophosphorus	-2
response to purine-containing compound	-2
response to calcium ion	-2
cellular response to metal ion	-2
RNA polymerase II transcription factor complex	-2
nuclear transcription factor complex	-2
cellular response to inorganic substance	-2
transcription coactivator activity	-2
response to metal ion	-2
DNA-binding transcription repressor activity, RNA polymerase II-specific	-2
positive regulation of nuclear-transcribed mRNA catabolic process, deadenylation-dependent decay	-2
regulation of nuclear-transcribed mRNA catabolic process, deadenylation-dependent decay	-2
p38MAPK cascade	-2
nuclear-transcribed mRNA catabolic process, deadenylation-dependent decay	-2
positive regulation of mRNA catabolic process	-2
nuclear-transcribed mRNA catabolic process	-2
regulation of mRNA catabolic process	-2
erythrocyte differentiation	-2
erythrocyte homeostasis	-2
negative regulation of cellular amide metabolic process	-2
myeloid cell homeostasis	-2
cytoplasmic ribonucleoprotein granule	-2
mRNA catabolic process	-2
ribonucleoprotein granule	-2
RNA catabolic process	-2
negative regulation of cellular catabolic process	-2
stress-activated MAPK cascade	-2
negative regulation of catabolic process	-2
stress-activated protein kinase signaling cascade	-2
RNA polymerase II activating transcription factor binding	-2
positive regulation of myeloid leukocyte differentiation	-2
SMAD binding	-2
activating transcription factor binding	-2
positive regulation of myeloid cell differentiation	-2
regulation of myeloid leukocyte differentiation	-2

transforming growth factor beta receptor signaling pathway	-2
RNA polymerase II transcription factor binding	-2
camera-type eye development	-2
vascular process in circulatory system	-2
positive regulation of ion transport	-2
regulation of small molecule metabolic process	-2
negative regulation of protein kinase activity	-2
negative regulation of kinase activity	-2
modification by host of symbiont morphology or physiology	-2
positive regulation of viral process	-2
modification of morphology or physiology of other organism involved in symbiotic interaction	-2
modification of morphology or physiology of other organism	-2
positive regulation of ERK1 and ERK2 cascade	-2
positive regulation of neuron apoptotic process	-2
learning or memory	-2
cognition	-2
positive regulation of tumor necrosis factor production	-2
positive regulation of tumor necrosis factor superfamily cytokine production	-2
leukocyte migration	-2
cellular response to hypoxia	-2
cellular response to decreased oxygen levels	-2
cellular response to oxygen levels	-2
response to hypoxia	-2
response to decreased oxygen levels	-2
T cell differentiation	-2
response to oxygen levels	-2
apoptotic mitochondrial changes	-2
regulation of extrinsic apoptotic signaling pathway	-2
extrinsic apoptotic signaling pathway	-2
cellular response to oxidative stress	-2
intrinsic apoptotic signaling pathway	-2
embryonic placenta development	-2
placenta development	-2
cytokine biosynthetic process	-2
cytokine metabolic process	-2
muscle cell proliferation	-2
response to toxic substance	-2
myelin sheath	-2
negative regulation of catalytic activity	-2.121320344
blood vessel morphogenesis	-2.121320344
response to mechanical stimulus	-2.236067977
nuclear chromatin	-2.236067977
response to inorganic substance	-2.236067977
skeletal muscle cell differentiation	-2.236067977
positive regulation of mRNA metabolic process	-2.236067977
cellular response to tumor necrosis factor	-2.236067977
regulation of mRNA metabolic process	-2.236067977
regulation of translation	-2.236067977
nucleobase-containing compound catabolic process	-2.236067977
aromatic compound catabolic process	-2.236067977
posttranscriptional regulation of gene expression	-2.236067977
positive regulation of leukocyte differentiation	-2.236067977

© 2021. Jin C et al. Published online at http://www.aginganddisease.org/EN/10.14336/AD.2021.0520

SUPPLEMENTARY DATA

cellular response to transforming growth factor beta stimulus	-2.236067977
positive regulation of hemopoiesis	-2.236067977
response to transforming growth factor beta	-2.236067977
regulation of leukocyte differentiation	-2.236067977
transmembrane receptor protein serine/threonine kinase signaling pathway	-2.236067977
protein heterodimerization activity	-2.236067977
negative regulation of inflammatory response	-2.236067977
negative regulation of defense response	-2.236067977
regulation of anatomical structure size	-2.236067977
negative regulation of transferase activity	-2.236067977
interaction with symbiont	-2.236067977
positive regulation of multi-organism process	-2.236067977
positive regulation of MAPK cascade	-2.236067977
positive regulation of neuron death	-2.236067977
regulation of epithelial cell proliferation	-2.236067977
epithelial cell proliferation	-2.236067977
response to wounding	-2.236067977
regulation of tumor necrosis factor production	-2.236067977
regulation of tumor necrosis factor superfamily cytokine production	-2.236067977
tumor necrosis factor production	-2.236067977
tumor necrosis factor superfamily cytokine production	-2.236067977
cytokine-mediated signaling pathway	-2.236067977
response to oxidative stress	-2.236067977
regulation of apoptotic signaling pathway	-2.236067977
axon development	-2.236067977
regulation of cellular catabolic process	-2.333333333
positive regulation of apoptotic process	-2.333333333
positive regulation of programmed cell death	-2.333333333
response to radiation	-2.449489743
transcription coregulator activity	-2.449489743
cellular response to hormone stimulus	-2.449489743
regulation of transcription from RNA polymerase II promoter in response to stress	-2.449489743
regulation of DNA-templated transcription in response to stress	-2.449489743
response to tumor necrosis factor	-2.449489743
homeostasis of number of cells	-2.449489743
regulation of cellular amide metabolic process	-2.449489743
cellular nitrogen compound catabolic process	-2.449489743
negative regulation of immune system process	-2.449489743
heterocycle catabolic process	-2.449489743
negative regulation of cell differentiation	-2.449489743
regulation of myeloid cell differentiation	-2.449489743
myeloid leukocyte differentiation	-2.449489743
negative regulation of response to external stimulus	-2.449489743
negative regulation of intracellular signal transduction	-2.449489743
regulation of viral process	-2.449489743
regulation of symbiosis, encompassing mutualism through parasitism	-2.449489743
viral process	-2.449489743
regulation of ERK1 and ERK2 cascade	-2.449489743
regulation of neuron apoptotic process	-2.449489743
epithelial cell differentiation	-2.449489743
developmental growth	-2.449489743
positive regulation of cytokine production	-2.449489743

© 2021. Jin C et al. Published online at http://www.aginganddisease.org/EN/10.14336/AD.2021.0520

cellular response to hypoxia	-2.449489743
apoptotic signaling pathway	-2.449489743
protein domain specific binding	-2.449489743
in utero embryonic development	-2.449489743
cytokine biosynthetic process	-2.449489743
positive regulation of cell death	-2.529822128
blood vessel development	-2.529822128
vasculature development	-2.529822128
cardiovascular system development	-2.529822128
response to organic cyclic compound	-2.529822128
response to lipopolysaccharide	-2.645751311
response to molecule of bacterial origin	-2.645751311
DNA-binding transcription activator activity, RNA polymerase II-specific	-2.645751311
response to hormone	-2.645751311
regulation of transcription from RNA polymerase II promoter in response to stress	-2.645751311
organic cyclic compound catabolic process	-2.645751311
regulation of hemopoiesis	-2.645751311
cellular response to growth factor stimulus	-2.645751311
regulation of inflammatory response	-2.645751311
ERK1 and ERK2 cascade	-2.645751311
symbiont process	-2.645751311
regulation of multi-organism process	-2.645751311
interspecies interaction between organisms	-2.645751311
regulation of MAPK cascade	-2.645751311
neuron apoptotic process	-2.645751311
regulation of neuron death	-2.645751311
myelin sheath	-2.645751311
RNA polymerase II proximal promoter sequence-specific DNA binding	-2.828427125
proximal promoter sequence-specific DNA binding	-2.828427125
skeletal muscle tissue development	-2.828427125
skeletal muscle organ development	-2.828427125
muscle organ development	-2.828427125
striated muscle tissue development	-2.828427125
muscle tissue development	-2.828427125
peptide metabolic process	-2.828427125
response to growth factor	-2.828427125
regulation of defense response	-2.828427125
negative regulation of protein phosphorylation	-2.828427125
negative regulation of protein modification process	-2.828427125
neuron death	-2.828427125
regulation of cytokine production	-2.828427125
regulation of cellular response to stress	-2.828427125
embryonic placenta development	-2.828427125
myeloid cell differentiation	-3
leukocyte differentiation	-3
regulation of response to external stimulus	-3
negative regulation of phosphorylation	-3
negative regulation of phosphorus metabolic process	-3
negative regulation of phosphate metabolic process	-3
MAPK cascade	-3
cytokine production	-3
negative regulation of phosphorylation	-3.050851079

positive regulation of cell death	-3.050851079
blood vessel development	-3.050851079
inflammatory response	-3.152963125
skeletal muscle tissue development	-3.16227766
muscle structure development	-3.16227766
regulation of tumor necrosis factor production	-3.207134903
transcription factor binding	-3.31662479
inflammatory response	-3.31662479
DNA-binding transcription factor activity, RNA polymerase II-specific	-3.464101615
interaction with symbiont	-3.605551275
apoptotic mitochondrial changes	-3.741657387
myeloid cell differentiation	-4.242640687
positive regulation of nuclear-transcribed mRNA catabolic process, deadenylation-dependent decay	-4.706787243
transcription factor AP-1 complex	-4.898979486
C1vsC0	
Terms	Z-score
DNA-binding transcription factor activity, RNA polymerase II-specific	3.605551275
transcription factor binding	3.16227766
myeloid cell differentiation	3.16227766
leukocyte differentiation	3.16227766
inflammatory response	3.16227766
RNA polymerase II proximal promoter sequence-specific DNA binding	3
regulation of response to external stimulus	3
proximal promoter sequence-specific DNA binding	3
muscle structure development	3
cytokine production	3
striated muscle tissue development	2.828427125
skeletal muscle tissue development	2.828427125
skeletal muscle organ development	2.828427125
regulation of transcription from RNA polymerase II promoter in response to stress	2.828427125
regulation of hemopoiesis	2.828427125
regulation of DNA-templated transcription in response to stress	2.828427125
regulation of defense response	2.828427125
regulation of cytokine production	2.828427125
regulation of cellular response to stress	2.828427125
peptide metabolic process	2.828427125
neuron death	2.828427125
muscle tissue development	2.828427125
muscle organ development	2.828427125
MAPK cascade	2.828427125
DNA-binding transcription activator activity, RNA polymerase II-specific	2.828427125
apoptotic signaling pathway	2.828427125
positive regulation of cell death	2.713602101
response to molecule of bacterial origin	2.645751311
response to lipopolysaccharide	2.645751311
response to hormone	2.645751311
regulation of neuron death	2.645751311
regulation of myeloid cell differentiation	2.645751311
regulation of inflammatory response	2.645751311
protein domain specific binding	2.645751311
neuron apoptotic process	2.645751311
negative regulation of phosphorylation	2.645751311

© 2021. Jin C et al. Published online at http://www.aginganddisease.org/EN/10.14336/AD.2021.0520

negative regulation of phosphorus metabolic process	2.645751311
negative regulation of phosphate metabolic process	2.645751311
negative regulation of immune system process	2.645751311
myeloid leukocyte differentiation	2.645751311
in utero embryonic development	2.645751311
chromatin binding	2.645751311
response to growth factor	2.529822128
transmembrane receptor protein serine/threonine kinase signaling pathway	2.449489743
translation	2.449489743
transcription coregulator activity	2.449489743
T cell activation	2.449489743
symbiont process	2.449489743
response to tumor necrosis factor	2.449489743
regulation of neuron apoptotic process	2.449489743
regulation of leukocyte differentiation	2.449489743
regulation of apoptotic signaling pathway	2.449489743
protein heterodimerization activity	2.449489743
positive regulation of cytokine production	2.449489743
peptide biosynthetic process	2.449489743
nuclear chromosome part	2.449489743
nuclear chromosome	2.449489743
nuclear chromatin	2.449489743
negative regulation of protein phosphorylation	2.449489743
negative regulation of protein modification process	2.449489743
negative regulation of intracellular signal transduction	2.449489743
negative regulation of cell proliferation	2.449489743
intrinsic apoptotic signaling pathway	2.449489743
interspecies interaction between organisms	2.449489743
homeostasis of number of cells	2.449489743
ERK1 and ERK2 cascade	2.449489743
chromatin	2.449489743
cellular response to hormone stimulus	2.449489743
vasculature development	2.333333333
response to organic cyclic compound	2.333333333
positive regulation of programmed cell death	2.333333333
positive regulation of apoptotic process	2.333333333
cellular response to growth factor stimulus	2.333333333
cardiovascular system development	2.333333333
blood vessel development	2.333333333
viral process	2.236067977
ubiquitin-like protein ligase binding	2.236067977
transcription factor complex	2.236067977
SMAD binding	2.236067977
skeletal muscle cell differentiation	2.236067977
RNA polymerase II transcription factor complex	2.236067977
response to wounding	2.236067977
response to transforming growth factor beta	2.236067977
response to radiation	2.236067977
response to mechanical stimulus	2.236067977
response to inorganic substance	2.236067977
reproductive system development	2.236067977
reproductive structure development	2.236067977

SUPPLEMENTARY DATA

regulation of viral process	2.236067977
regulation of symbiosis, encompassing mutualism through parasitism	2.236067977
regulation of myeloid leukocyte differentiation	2.236067977
regulation of multi-organism process	2.236067977
regulation of mRNA metabolic process	2.236067977
regulation of ERK1 and ERK2 cascade	2.236067977
positive regulation of neuron death	2.236067977
positive regulation of mRNA metabolic process	2.236067977
positive regulation of leukocyte differentiation	2.236067977
positive regulation of hemopoiesis	2.236067977
placenta development	2.236067977
organic cyclic compound catabolic process	2.236067977
nucleobase-containing compound catabolic process	2.236067977
nuclear transcription factor complex	2.236067977
negative regulation of response to external stimulus	2.236067977
heterocycle catabolic process	2.236067977
extrinsic apoptotic signaling pathway	2.236067977
epithelial cell proliferation	2.236067977
embryonic placenta development	2.236067977
embryonic organ development	2.236067977
DNA-binding transcription repressor activity, RNA polymerase II-specific	2.236067977
cytokine-mediated signaling pathway	2.236067977
cellular response to tumor necrosis factor	2.236067977
cellular response to transforming growth factor beta stimulus	2.236067977
cellular nitrogen compound catabolic process	2.236067977
aromatic compound catabolic process	2.236067977
regulation of cellular catabolic process	2.121320344
epithelial cell differentiation	2.121320344
ubiquitin protein ligase binding	2
tumor necrosis factor superfamily cytokine production	2
tumor necrosis factor production	2
transforming growth factor beta receptor signaling pathway	2
transcription factor AP-1 complex	2
transcription coactivator activity	2
temperature homeostasis	2
T cell differentiation	2
stress-activated protein kinase signaling cascade	2
stress-activated MAPK cascade	2
RNA catabolic process	2
response to purine-containing compound	2
response to organophosphorus	2
response to metal ion	2
response to interleukin-1	2
response to endoplasmic reticulum stress	2
response to cAMP	2
response to calcium ion	2
regulation of tumor necrosis factor superfamily cytokine production	2
regulation of tumor necrosis factor production	2
regulation of translation	2
regulation of nuclear-transcribed mRNA catabolic process, deadenylation-dependent decay	2
regulation of mRNA catabolic process	2
regulation of intrinsic apoptotic signaling pathway	2

© 2021. Jin C et al. Published online at http://www.aginganddisease.org/EN/10.14336/AD.2021.0520

regulation of extrinsic apoptotic signaling pathway	2
reactive oxygen species metabolic process	2
positive regulation of response to external stimulus	2
positive regulation of nuclear-transcribed mRNA catabolic process, deadenylation-dependent decay	2
positive regulation of neuron apoptotic process	2
positive regulation of myeloid leukocyte differentiation	2
positive regulation of myeloid cell differentiation	2
positive regulation of mRNA catabolic process	2
p38MAPK cascade	2
osteoclast differentiation	2
nuclear-transcribed mRNA catabolic process, deadenylation-dependent decay	2
nuclear-transcribed mRNA catabolic process	2
negative regulation of transcription from RNA polymerase II promoter in response to stress	2
negative regulation of inflammatory response	2
negative regulation of defense response	2
negative regulation of cellular catabolic process	2
negative regulation of catabolic process	2
negative regulation of apoptotic signaling pathway	2
myeloid cell homeostasis	2
mRNA catabolic process	2
mRNA binding	2
intrinsic apoptotic signaling pathway in response to DNA damage	2
interaction with symbiont	2
heat shock protein binding	2
fat cell differentiation	2
erythrocyte homeostasis	2
erythrocyte differentiation	2
cytosolic part	2
cytokine metabolic process	2
cytokine biosynthetic process	2
cellular response to oxidative stress	2
cellular response to molecule of bacterial origin	2
cellular response to metal ion	2
cellular response to lipopolysaccharide	2
cellular response to interleukin-1	2
cellular response to inorganic substance	2
cellular response to calcium ion	2
cellular response to biotic stimulus	2
apoptotic mitochondrial changes	2
C2vsC0	
Terms	Z-score
transcription factor binding	2.828427125
protein domain specific binding	2.645751311
positive regulation of cell death	2.449489743
synapse organization	2.449489743
regulation of anatomical structure size	2.449489743
cell morphogenesis involved in neuron differentiation	2.449489743
neuron projection morphogenesis	2.449489743
plasma membrane bounded cell projection morphogenesis	2.449489743
cell projection morphogenesis	2.449489743
cell part morphogenesis	2.449489743
protein kinase binding	2.449489743

© 2021. Jin C et al. Published online at http://www.aginganddisease.org/EN/10.14336/AD.2021.0520

positive regulation of apoptotic process	2.236067977
positive regulation of programmed cell death	2.236067977
regulation of cell morphogenesis involved in differentiation	2.236067977
regulation of cellular component size	2.236067977
axonogenesis	2.236067977
establishment of organelle localization	2.236067977
axon development	2.236067977
regulation of cell morphogenesis	2.236067977
organelle localization	2.236067977
regulation of neuron projection development	2.236067977
negative regulation of catalytic activity	2.236067977
regulation of growth	2.236067977
mRNA metabolic process	2.236067977
inflammatory response	2.236067977
regulation of hemopoiesis	2
positive regulation of cell morphogenesis involved in differentiation	2
regulation of axonogenesis	2
vesicle-mediated transport in synapse	2
regulation of synapse organization	2
regulation of synapse structure or activity	2
positive regulation of neuron projection development	2
postsynaptic density	2
asymmetric synapse	2
neuron to neuron synapse	2
microtubule	2
postsynaptic specialization	2
regulation of cell growth	2
positive regulation of neuron differentiation	2
positive regulation of cell projection organization	2
myelin sheath	2
regulation of mRNA metabolic process	2
regulation of cellular amide metabolic process	2
C3vsC0	
Terms	Z-score
cytosolic part	7.141428429
ribosome	7
ribosomal subunit	6.92820323
amide biosynthetic process	6.861372412
cytosolic ribosome	6.8556546
translation	6.788225099
peptide biosynthetic process	6.788225099
structural constituent of ribosome	6.708203932
structural molecule activity	6.428571429
peptide metabolic process	6.414269806
large ribosomal subunit	5.196152423
cytosolic large ribosomal subunit	5.099019514
ribonucleoprotein complex biogenesis	4.706787243
small ribosomal subunit	4.69041576
cytosolic small ribosomal subunit	4.582575695
ribosome biogenesis	4.490731195
purine ribonucleoside monophosphate metabolic process	4.242640687
purine nucleoside monophosphate metabolic process	4.242640687

© 2021. Jin C et al. Published online at http://www.aginganddisease.org/EN/10.14336/AD.2021.0520

ribonucleoside monophosphate metabolic process	4.242640687
nucleoside monophosphate metabolic process	4.242640687
drug metabolic process	4.157609203
ATP metabolic process	4.123105626
nucleoside triphosphate metabolic process	4.024922359
purine ribonucleotide metabolic process	4.024922359
ribonucleotide metabolic process	4.024922359
purine nucleotide metabolic process	4.024922359
ribose phosphate metabolic process	4.024922359
purine-containing compound metabolic process	4.024922359
nucleobase-containing small molecule metabolic process	3.961773867
purine ribonucleoside triphosphate metabolic process	3.900067476
ribonucleoside triphosphate metabolic process	3.900067476
purine nucleoside triphosphate metabolic process	3.900067476
cytoplasmic translation	3.872983346
ribosomal small subunit biogenesis	3.872983346
ribonucleoprotein complex assembly	3.872983346
ribonucleoprotein complex subunit organization	3.872983346
nucleoside phosphate metabolic process	3.837612894
ncRNA processing	3.741657387
ncRNA metabolic process	3.741657387
rRNA binding	3.741657387
organic cyclic compound catabolic process	3.709704134
nucleotide metabolic process	3.709704134
postsynaptic specialization	3.709704134
rRNA processing	3.605551275
rRNA metabolic process	3.605551275
purine nucleoside monophosphate biosynthetic process	3.605551275
purine ribonucleoside monophosphate biosynthetic process	3.605551275
ribonucleoside monophosphate biosynthetic process	3.605551275
nucleoside monophosphate biosynthetic process	3.605551275
cofactor biosynthetic process	3.605551275
generation of precursor metabolites and energy	3.605551275
ribosome assembly	3.605551275
postsynaptic density	3.577708764
asymmetric synapse	3.577708764
neuron to neuron synapse	3.577708764
nucleoside diphosphate metabolic process	3.464101615
ATP biosynthetic process	3.464101615
nucleoside phosphate catabolic process	3.464101615
coenzyme biosynthetic process	3.464101615
organophosphate catabolic process	3.464101615
monosaccharide metabolic process	3.464101615
cellular nitrogen compound catabolic process	3.441236008
heterocycle catabolic process	3.441236008
aromatic compound catabolic process	3.441236008
cofactor metabolic process	3.441236008
negative regulation of intracellular signal transduction	3.441236008
purine ribonucleotide biosynthetic process	3.356585567
ribonucleotide biosynthetic process	3.356585567
ribose phosphate biosynthetic process	3.356585567
purine nucleotide biosynthetic process	3.356585567

SUPPLEMENTARY DATA

purine-containing compound biosynthetic process	3.356585567
nucleotide biosynthetic process	3.356585567
nucleoside phosphate biosynthetic process	3.356585567
polysome	3.31662479
ADP metabolic process	3.31662479
nucleoside diphosphate phosphorylation	3.31662479
purine nucleoside diphosphate metabolic process	3.31662479
purine ribonucleoside diphosphate metabolic process	3.31662479
nucleotide phosphorylation	3.31662479
ribonucleoside diphosphate metabolic process	3.31662479
nucleotide catabolic process	3.31662479
nucleobase-containing compound catabolic process	3.299831646
homeostasis of number of cells	3.299831646
carbohydrate catabolic process	3.207134903
purine ribonucleoside triphosphate biosynthetic process	3.207134903
purine nucleoside triphosphate biosynthetic process	3.207134903
ribonucleoside triphosphate biosynthetic process	3.207134903
nucleoside triphosphate biosynthetic process	3.207134903
nucleobase-containing small molecule biosynthetic process	3.207134903
coenzyme metabolic process	3.207134903
polysomal ribosome	3.16227766
glycolytic process	3.16227766
ATP generation from ADP	3.16227766
pyruvate biosynthetic process	3.16227766
nicotinamide nucleotide biosynthetic process	3.16227766
pyridine nucleotide biosynthetic process	3.16227766
pyridine-containing compound biosynthetic process	3.16227766
hexose metabolic process	3.16227766
response to interferon-gamma	3.16227766
cytokine activity	3.16227766
carbohydrate metabolic process	3.130495168
regulation of neuron death	3.130495168
neuron death	3.127716211
postsynapse	3.127716211
regulation of ERK1 and ERK2 cascade	3.050851079
ERK1 and ERK2 cascade	3.050851079
myeloid cell homeostasis	3.050851079
glucose metabolic process	3
ribosomal small subunit assembly	3
ribosomal large subunit biogenesis	3
apoptotic signaling pathway	3
carbohydrate derivative biosynthetic process	2.98240454
amide binding	2.98240454
myeloid cell differentiation	2.98240454
pyruvate metabolic process	2.886751346
nicotinamide nucleotide metabolic process	2.886751346
pyridine nucleotide metabolic process	2.886751346
pyridine-containing compound metabolic process	2.886751346
oxidoreduction coenzyme metabolic process	2.886751346
intrinsic apoptotic signaling pathway in response to DNA damage	2.886751346
erythrocyte homeostasis	2.886751346
negative regulation of apoptotic signaling pathway	2.840187787

© 2021. Jin C et al. Published online at http://www.aginganddisease.org/EN/10.14336/AD.2021.0520

organophosphate biosynthetic process	2.836832573
regulation of MAPK cascade	2.836832573
monocarboxylic acid biosynthetic process	2.828427125
carbohydrate biosynthetic process	2.828427125
vacuolar membrane	2.828427125
vacuolar part	2.828427125
response to tumor necrosis factor	2.828427125
regulation of apoptotic signaling pathway	2.828427125
mitochondrial outer membrane	2.828427125
outer membrane	2.828427125
organelle outer membrane	2.828427125
peptide binding	2.828427125
small molecule biosynthetic process	2.785430073
MAPK cascade	2.710687383
regulation of cytokine production	2.69407953
positive regulation of cell death	2.69407953
neuron apoptotic process	2.667891875
monosaccharide biosynthetic process	2.645751311
cellular response to interferon-gamma	2.645751311
regulation of response to external stimulus	2.611164839
regulation of cellular response to stress	2.558408596
granulocyte chemotaxis	2.529822128
granulocyte migration	2.529822128
positive regulation of ERK1 and ERK2 cascade	2.529822128
negative regulation of intrinsic apoptotic signaling pathway	2.529822128
lysosomal membrane	2.449489743
lytic vacuole membrane	2.449489743
chemokine receptor binding	2.449489743
regulation of fibroblast proliferation	2.449489743
fibroblast proliferation	2.449489743
positive regulation of cell migration	2.449489743
positive regulation of cell motility	2.449489743
positive regulation of cellular component movement	2.449489743
positive regulation of locomotion	2.449489743
negative regulation of transport	2.449489743
vacuole	2.414039396
inflammatory response	2.400980192
monocarboxylic acid metabolic process	2.400396793
negative regulation of response to external stimulus	2.357022604
intrinsic apoptotic signaling pathway	2.357022604
neutrophil chemotaxis	2.333333333
neutrophil migration	2.333333333
positive regulation of myeloid cell differentiation	2.333333333
regulation of myeloid leukocyte differentiation	2.333333333
negative regulation of neuron death	2.323790008
regulation of neuron apoptotic process	2.323790008
regulation of myeloid cell differentiation	2.323790008
regulation of intrinsic apoptotic signaling pathway	2.309401077
carboxylic acid biosynthetic process	2.293658555
organic acid biosynthetic process	2.293658555
leukocyte migration	2.293658555
positive regulation of cytokine production	2.293658555

lysosome	2.267786838
lytic vacuole	2.267786838
peptide secretion	2.267786838
positive regulation of programmed cell death	2.267786838
cytokine production	2.263009527
maturation of SSU-rRNA	2.236067977
large ribosomal subunit rRNA binding	2.236067977
cellular aldehyde metabolic process	2.236067977
gluconeogenesis	2.236067977
hexose biosynthetic process	2.236067977
regulation of ATP metabolic process	2.236067977
ribosomal large subunit assembly	2.236067977
chemokine activity	2.236067977
CCR chemokine receptor binding	2.236067977
lymphocyte chemotaxis	2.236067977
response to chemokine	2.236067977
cellular response to chemokine	2.236067977
positive regulation of fibroblast proliferation	2.236067977
leukocyte chemotaxis	2.182820625
myeloid leukocyte differentiation	2.138089935
leukocyte differentiation	2.132007164
positive regulation of myeloid leukocyte differentiation	2.121320344
osteoclast differentiation	2.121320344
regulation of peptide secretion	2.116950987
protein secretion	2.116950987
positive regulation of apoptotic process	2.116950987
mononuclear cell migration	2.110579412
myeloid leukocyte migration	2.064741605
regulation of hemopoiesis	2.064741605
regulation of establishment of protein localization	2.04264872
regulation of peptide transport	2.04264872
maturation of SSU-rRNA from tricistronic rRNA transcript (SSU-rRNA, 5.8S rRNA, LSU-rRNA)	2
regulation of glycolytic process	2
regulation of nucleotide catabolic process	2
regulation of carbohydrate catabolic process	2
regulation of coenzyme metabolic process	2
cellular response to interleukin-4	2
response to interleukin-4	2
eosinophil chemotaxis	2
eosinophil migration	2
BH domain binding	2
myeloid leukocyte cytokine production	2
positive regulation of cytokine production involved in immune response	2
C4vsC0	
Terms	Z-score
modulation of chemical synaptic transmission	3.16227766
regulation of trans-synaptic signaling	3.16227766
dendrite	3.16227766
dendritic tree	3.16227766
positive regulation of synaptic transmission	3
axon	3
inorganic molecular entity transmembrane transporter activity	3

© 2021. Jin C et al. Published online at http://www.aginganddisease.org/EN/10.14336/AD.2021.0520

glial cell differentiation	2.828427125
synapse organization	2.828427125
inorganic cation transmembrane transporter activity	2.828427125
cation transmembrane transporter activity	2.828427125
postsynapse	2.828427125
behavior	2.713602101
learning or memory	2.645751311
cognition	2.645751311
cell projection morphogenesis	2.645751311
cell part morphogenesis	2.645751311
axon part	2.645751311
amide binding	2.645751311
peptide metabolic process	2.645751311
monovalent inorganic cation transmembrane transporter activity	2.645751311
response to inorganic substance	2.645751311
mitochondrial membrane	2.645751311
mitochondrial envelope	2.645751311
generation of precursor metabolites and energy	2.645751311
positive regulation of cell death	2.645751311
regulation of MAPK cascade	2.645751311
MAPK cascade	2.645751311
glutamatergic synapse	2.645751311
presynapse	2.645751311
gliogenesis	2.529822128
neuronal cell body	2.529822128
negative regulation of nervous system development	2.449489743
regulation of synapse organization	2.449489743
regulation of synapse structure or activity	2.449489743
regulation of synaptic plasticity	2.449489743
cell morphogenesis involved in neuron differentiation	2.449489743
neuron projection morphogenesis	2.449489743
plasma membrane bounded cell projection morphogenesis	2.449489743
distal axon	2.449489743
peptide binding	2.449489743
protein homooligomerization	2.449489743
negative regulation of phosphorylation	2.449489743
negative regulation of phosphorus metabolic process	2.449489743
negative regulation of phosphate metabolic process	2.449489743
protein complex oligomerization	2.449489743
neurotransmitter transport	2.449489743
anion transport	2.449489743
response to toxic substance	2.449489743
positive regulation of transmembrane transport	2.449489743
mitochondrial inner membrane	2.449489743
organelle inner membrane	2.449489743
small molecule biosynthetic process	2.449489743
positive regulation of apoptotic process	2.449489743
positive regulation of programmed cell death	2.449489743
regulation of ERK1 and ERK2 cascade	2.449489743
ERK1 and ERK2 cascade	2.449489743
regulation of transmembrane transport	2.333333333
cellular metal ion homeostasis	2.333333333

© 2021. Jin C et al. Published online at http://www.aginganddisease.org/EN/10.14336/AD.2021.0520

SUPPLEMENTARY DATA

cellular cation homeostasis	2.333333333
cellular ion homeostasis	2.333333333
metal ion homeostasis	2.333333333
cation homeostasis	2.333333333
inorganic ion homeostasis	2.333333333
drug metabolic process	2.333333333
glial cell development	2.236067977
negative regulation of neurogenesis	2.236067977
negative regulation of cell development	2.236067977
long-term synaptic potentiation	2.236067977
regulation of cell morphogenesis	2.236067977
growth cone	2.236067977
site of polarized growth	2.236067977
aging	2.236067977
sulfur compound binding	2.236067977
negative regulation of protein phosphorylation	2.236067977
import across plasma membrane	2.236067977
metal ion transmembrane transporter activity	2.236067977
positive regulation of cation transmembrane transport	2.236067977
positive regulation of ion transmembrane transport	2.236067977
ATP metabolic process	2.236067977
purine ribonucleoside monophosphate metabolic process	2.236067977
purine ribonucleoside triphosphate metabolic process	2.236067977
purine nucleoside monophosphate metabolic process	2.236067977
ribonucleoside monophosphate metabolic process	2.236067977
ribonucleoside triphosphate metabolic process	2.236067977
purine nucleoside triphosphate metabolic process	2.236067977
nucleoside monophosphate metabolic process	2.236067977
nucleoside triphosphate metabolic process	2.236067977
carboxylic acid biosynthetic process	2.236067977
organic acid biosynthetic process	2.236067977
purine ribonucleotide metabolic process	2.236067977
ribonucleotide metabolic process	2.236067977
purine nucleotide metabolic process	2.236067977
ribose phosphate metabolic process	2.236067977
purine-containing compound metabolic process	2.236067977
cofactor metabolic process	2.236067977
positive regulation of MAPK cascade	2.236067977
synaptic membrane	2.236067977
regulation of cellular protein localization	2.236067977
regulation of cation transmembrane transport	2.121320344
regulation of ion transmembrane transport	2.121320344
regulation of neurotransmitter levels	2.121320344
cellular divalent inorganic cation homeostasis	2.121320344
divalent inorganic cation homeostasis	2.121320344
perinuclear region of cytoplasm	2.121320344
cell body	2.110579412
regulation of ion transport	2.110579412
astrocyte differentiation	2
oligodendrocyte differentiation	2
memory	2
regulation of calcium ion transmembrane transport	2

© 2021. Jin C et al. Published online at http://www.aginganddisease.org/EN/10.14336/AD.2021.0520

postsynapse organization	2
neurotransmitter uptake	2
sodium ion transmembrane transporter activity	2
dendritic spine	2
neuron spine	2
response to light stimulus	2
carboxylic acid transport	2
organic acid transport	2
detoxification	2
cellular response to metal ion	2
cellular response to inorganic substance	2
response to metal ion	2
positive regulation of ion transmembrane transporter activity	2
positive regulation of transporter activity	2
mitochondrial transport	2
energy derivation by oxidation of organic compounds	2
monocarboxylic acid biosynthetic process	2
antioxidant activity	2
cellular lipid catabolic process	2
lipid catabolic process	2
positive regulation of ERK1 and ERK2 cascade	2
regulation of blood pressure	2
C5vsC0	
Terms	Z-score
defense response to other organism	3.464101615
response to virus	3.31662479
defense response to virus	3.16227766
interspecies interaction between organisms	3.16227766
regulation of cytokine production	3
cytokine production	3
viral process	2.828427125
symbiont process	2.828427125
cytokine-mediated signaling pathway	2.828427125
regulation of viral process	2.645751311
regulation of symbiosis, encompassing mutualism through parasitism	2.645751311
regulation of multi-organism process	2.645751311
response to interferon-gamma	2.645751311
regulation of defense response	2.645751311
positive regulation of immune response	2.645751311
negative regulation of viral process	2.449489743
negative regulation of multi-organism process	2.449489743
response to interferon-beta	2.449489743
negative regulation of cellular component organization	2.449489743
positive regulation of cytokine production	2.449489743
negative regulation of cell proliferation	2.449489743
regulation of viral life cycle	2.236067977
viral life cycle	2.236067977
cellular response to interferon-gamma	2.236067977
G protein-coupled receptor binding	2.236067977
positive regulation of hydrolase activity	2.236067977
response to type I interferon	2.236067977
regulation of innate immune response	2.236067977

regulation of immune effector process	2.236067977
myeloid cell homeostasis	2.236067977
homeostasis of number of cells	2.236067977
myeloid cell differentiation	2.236067977
negative regulation of viral genome replication	2
negative regulation of viral life cycle	2
regulation of viral genome replication	2
viral genome replication	2
response to interferon-alpha	2
CCR chemokine receptor binding	2
monocyte chemotaxis	2
chemokine receptor binding	2
response to chemokine	2
cellular response to chemokine	2
mononuclear cell migration	2
regulation of leukocyte chemotaxis	2
cellular response to tumor necrosis factor	2
response to tumor necrosis factor	2
leukocyte chemotaxis	2
cell chemotaxis	2
regulation of ERK1 and ERK2 cascade	2
cytokine receptor binding	2
ERK1 and ERK2 cascade	2
regulation of epithelial cell proliferation	2
response to molecule of bacterial origin	2
epithelial cell proliferation	2
type I interferon signaling pathway	2
cellular response to type I interferon	2
positive regulation of innate immune response	2
positive regulation of defense response	2
positive regulation of leukocyte mediated cytotoxicity	2
positive regulation of cell killing	2
regulation of leukocyte mediated cytotoxicity	2
regulation of cell killing	2
leukocyte mediated cytotoxicity	2
cell killing	2
regulation of adaptive immune response	2
positive regulation of immune effector process	2
lymphocyte mediated immunity	2
erythrocyte differentiation	2
erythrocyte homeostasis	2
regulation of myeloid cell differentiation	2
regulation of hemopoiesis	2

Supplementary Table 3. Functional enrichment results of DEGs for HAM in whole brain.

Substance and energy metabolism	
Terms	Z-score
amide biosynthetic process	6.32455532
peptide biosynthetic process	6.32455532

© 2021. Jin C et al. Published online at http://www.aginganddisease.org/EN/10.14336/AD.2021.0520

peptide metabolic process	5.813776741
organic cyclic compound catabolic process	4.024922359
drug metabolic process	3.77964473
aromatic compound catabolic process	3.771236166
cellular nitrogen compound catabolic process	3.771236166
heterocycle catabolic process	3.771236166
nucleobase-containing compound catabolic process	3.638034376
nucleoside monophosphate metabolic process	3.638034376
purine nucleoside monophosphate metabolic process	3.638034376
purine ribonucleoside monophosphate metabolic process	3.638034376
ribonucleoside monophosphate metabolic process	3.638034376
ATP metabolic process	3.5
nucleoside triphosphate metabolic process	3.441236008
purine nucleotide metabolic process	3.441236008
purine ribonucleotide metabolic process	3.441236008
purine-containing compound metabolic process	3.441236008
ribonucleotide metabolic process	3.441236008
ribose phosphate metabolic process	3.441236008
cofactor biosynthetic process	3.31662479
nucleoside diphosphate metabolic process	3.31662479
nucleoside monophosphate biosynthetic process	3.31662479
nucleoside phosphate catabolic process	3.31662479
organophosphate catabolic process	3.31662479
purine nucleoside monophosphate biosynthetic process	3.31662479
purine ribonucleoside monophosphate biosynthetic process	3.31662479
ribonucleoside monophosphate biosynthetic process	3.31662479
purine nucleoside triphosphate metabolic process	3.299831646
purine ribonucleoside triphosphate metabolic process	3.299831646
ribonucleoside triphosphate metabolic process	3.299831646
nucleobase-containing small molecule metabolic process	3.273268354
nucleoside phosphate metabolic process	3.273268354
generation of precursor metabolites and energy	3.207134903
ADP metabolic process	3.16227766
ATP biosynthetic process	3.16227766
coenzyme biosynthetic process	3.16227766
monosaccharide metabolic process	3.16227766
nucleotide catabolic process	3.16227766
purine nucleoside diphosphate metabolic process	3.16227766
purine ribonucleoside diphosphate metabolic process	3.16227766

© 2021. Jin C et al. Published online at http://www.aginganddisease.org/EN/10.14336/AD.2021.0520

SUPPLEMENTARY DATA

ribonucleoside diphosphate metabolic process	3.16227766
nucleotide metabolic process	3.130495168
carbohydrate catabolic process	3.050851079
coenzyme metabolic process	3.050851079
nucleobase-containing small molecule biosynthetic process	3.050851079
nucleoside phosphate biosynthetic process	3.050851079
nucleotide biosynthetic process	3.050851079
purine nucleotide biosynthetic process	3.050851079
purine ribonucleotide biosynthetic process	3.050851079
purine-containing compound biosynthetic process	3.050851079
ribonucleotide biosynthetic process	3.050851079
ribose phosphate biosynthetic process	3.050851079
ATP generation from ADP	3
glycolytic process	3
hexose metabolic process	3
nicotinamide nucleotide biosynthetic process	3
pyridine nucleotide biosynthetic process	3
pyridine-containing compound biosynthetic process	3
pyruvate biosynthetic process	3
cofactor metabolic process	2.98240454
nucleoside triphosphate biosynthetic process	2.886751346
purine nucleoside triphosphate biosynthetic process	2.886751346
purine ribonucleoside triphosphate biosynthetic process	2.886751346
ribonucleoside triphosphate biosynthetic process	2.886751346
carbohydrate metabolic process	2.828427125
glucose metabolic process	2.828427125
nicotinamide nucleotide metabolic process	2.713602101
oxidoreduction coenzyme metabolic process	2.713602101
pyridine nucleotide metabolic process	2.713602101
pyridine-containing compound metabolic process	2.713602101
pyruvate metabolic process	2.713602101
organophosphate biosynthetic process	2.523573073
monocarboxylic acid biosynthetic process	2.5
monosaccharide biosynthetic process	2.449489743
monocarboxylic acid metabolic process	2.357022604
small molecule biosynthetic process	2.353393622
cellular aldehyde metabolic process	2.236067977
gluconeogenesis	2.236067977
hexose biosynthetic process	2.236067977

© 2021. Jin C et al. Published online at http://www.aginganddisease.org/EN/10.14336/AD.2021.0520

negative regulation of lipid metabolic process	2.236067977
regulation of ATP biosynthetic process	2
regulation of carbohydrate catabolic process	2
regulation of coenzyme metabolic process	2
regulation of glycolytic process	2
regulation of nucleotide catabolic process	2
Chemotaxis	
Terms	Z-score
CCR chemokine receptor binding	2.236067977
cell chemotaxis	2.293658555
chemokine activity	2.449489743
chemokine receptor binding	2.645751311
chemokine-mediated signaling pathway	2
chemotaxis	2.04264872
eosinophil chemotaxis	2
eosinophil migration	2
granulocyte chemotaxis	2.529822128
granulocyte migration	2.529822128
leukocyte chemotaxis	2.357022604
leukocyte migration	2.449489743
lymphocyte chemotaxis	2.449489743
mononuclear cell migration	2.110579412
myeloid leukocyte migration	2.064741605
neutrophil chemotaxis	2.333333333
neutrophil migration	2.333333333
positive regulation of blood vessel endothelial cell migration	2
positive regulation of cell migration	2.745625892
positive regulation of cell motility	2.745625892
positive regulation of cellular component movement	2.745625892
positive regulation of locomotion	2.745625892
regulation of leukocyte migration	2.138089935
taxis	2.19089023
Ribosome activity	
Terms	Z-score
cytoplasmic translation	3.16227766
cytosolic large ribosomal subunit	4
cytosolic ribosome	5.830951895
cytosolic small ribosomal subunit	4.242640687
large ribosomal subunit	4.123105626

© 2021. Jin C et al. Published online at http://www.aginganddisease.org/EN/10.14336/AD.2021.0520

large ribosomal subunit rRNA binding	2
polysomal ribosome	2.236067977
polysome	2.449489743
ribonucleoprotein complex assembly	3.16227766
ribonucleoprotein complex biogenesis	3.900067476
ribonucleoprotein complex subunit organization	3.16227766
ribosomal small subunit assembly	2.645751311
ribosomal small subunit biogenesis	3.31662479
ribosomal subunit	5.916079783
ribosome	6
ribosome assembly	3
ribosome biogenesis	3.771236166
rRNA binding	3
small ribosomal subunit	4.358898944
structural constituent of ribosome	5.830951895
translation	6.32455532
Cell survival	
Terms	Z-score
regulation of neuron death	3.273268354
apoptotic signaling pathway	3.265986324
neuron death	3.265986324
positive regulation of cell death	3.023715784
negative regulation of apoptotic signaling pathway	2.840187787
neuron apoptotic process	2.828427125
regulation of apoptotic signaling pathway	2.828427125
intrinsic apoptotic signaling pathway in response to DNA damage	2.713602101
negative regulation of neuron death	2.667891875
positive regulation of programmed cell death	2.6
intrinsic apoptotic signaling pathway	2.5
regulation of neuron apoptotic process	2.5
positive regulation of apoptotic process	2.449489743
regulation of leukocyte apoptotic process	2.334868926
negative regulation of intrinsic apoptotic signaling pathway	2.333333333
regulation of intrinsic apoptotic signaling pathway	2.110579412
Cellular response to stimulus	
Terms	Z-score
cellular response to chemokine	2.236067977
cellular response to interferon-gamma	2.645751311
cellular response to lipoprotein particle stimulus	2

© 2021. Jin C et al. Published online at http://www.aginganddisease.org/EN/10.14336/AD.2021.0520

SUPPLEMENTARY DATA

DNA damage response, signal transduction by p53 class mediator	2
negative regulation of response to external stimulus	2.828427125
negative regulation of response to wounding	2.449489743
regulation of cellular response to stress	2.683281573
regulation of response to external stimulus	3.211586168
regulation of response to wounding	2.828427125
response to chemokine	2.236067977
response to interferon-gamma	3.16227766
response to lipoprotein particle	2
response to tumor necrosis factor	2.828427125
response to wounding	4.146139914
Coagulation and hemostasis	
Terms	Z-score
blood coagulation	2.713602101
coagulation	2.713602101
hemostasis	2.713602101
negative regulation of blood coagulation	2.236067977
negative regulation of coagulation	2.236067977
negative regulation of hemostasis	2.236067977
negative regulation of wound healing	2.449489743
platelet activation	2.121320344
regulation of blood coagulation	2.449489743
regulation of coagulation	2.449489743
regulation of hemostasis	2.449489743
regulation of platelet activation	2
regulation of wound healing	2.645751311
wound healing	3.5
Immunity and inflammation	
Terms	Z-score
inflammatory response	2.743977362
leukocyte differentiation	2.558408596
myeloid cell differentiation	3.441236008
myeloid leukocyte differentiation	2.496150883
negative regulation of immune system process	3.130495168
positive regulation of leukocyte differentiation	3.271651525
positive regulation of myeloid cell differentiation	2.333333333
positive regulation of myeloid leukocyte differentiation	2.121320344
regulation of leukocyte differentiation	2.138089935
regulation of myeloid cell differentiation	2.672612419

© 2021. Jin C et al. Published online at http://www.aginganddisease.org/EN/10.14336/AD.2021.0520

regulation of myeloid leukocyte differentiation	2.333333333
Cytokine secretion	
Terms	Z-score
cytokine activity	3.31662479
cytokine production	2.263009527
cytokine-mediated signaling pathway	2.138089935
myeloid leukocyte cytokine production	2
positive regulation of cytokine production	2.293658555
positive regulation of cytokine production involved in immune response	2
regulation of cytokine production	2.69407953

Supplementary Table 4. Functional enrichment results of DEGs for age-associated microglia in subependymal ventricular zone.

Substance and energy metabolism	
Terms	Z-score
ADP metabolic process	3
ATP metabolic process	3.741657387
carbohydrate metabolic process	3.638034376
coenzyme metabolic process	3.16227766
cofactor metabolic process	4.358898944
drug metabolic process	5
generation of precursor metabolites and energy	3.464101615
glucose metabolic process	2.828427125
hexose metabolic process	3
monocarboxylic acid metabolic process	4
monosaccharide metabolic process	3
neurotransmitter metabolic process	2.449489743
nicotinamide nucleotide metabolic process	3
nitric oxide metabolic process	2.449489743
nucleobase-containing small molecule metabolic process	4
nucleoside diphosphate metabolic process	3.16227766
nucleoside monophosphate metabolic process	3.741657387
nucleoside phosphate metabolic process	4
nucleoside triphosphate metabolic process	4
nucleotide metabolic process	3.872983346
oxidoreduction coenzyme metabolic process	3
positive regulation of reactive oxygen species metabolic process	2.645751311

purine nucleoside diphosphate metabolic process	3
purine nucleoside monophosphate metabolic process	3.741657387
purine nucleoside triphosphate metabolic process	3.872983346
purine nucleotide metabolic process	3.872983346
purine ribonucleoside diphosphate metabolic process	3
purine ribonucleoside monophosphate metabolic process	3.741657387
purine ribonucleoside triphosphate metabolic process	3.872983346
purine ribonucleotide metabolic process	3.872983346
purine-containing compound metabolic process	3.872983346
pyridine nucleotide metabolic process	3
pyridine-containing compound metabolic process	3
pyruvate metabolic process	3
reactive nitrogen species metabolic process	2.449489743
reactive oxygen species metabolic process	3.872983346
regulation of ATP metabolic process	2.236067977
regulation of coenzyme metabolic process	2
regulation of cofactor metabolic process	2.236067977
regulation of nucleotide metabolic process	2.236067977
regulation of purine nucleotide metabolic process	2.236067977
regulation of reactive oxygen species metabolic process	3.31662479
ribonucleoside diphosphate metabolic process	3
ribonucleoside monophosphate metabolic process	3.741657387
ribonucleoside triphosphate metabolic process	3.872983346
ribonucleotide metabolic process	3.872983346
ribose phosphate metabolic process	3.872983346
aromatic compound catabolic process	3.900067476
carbohydrate catabolic process	2.529822128
cellular nitrogen compound catabolic process	3.900067476
heterocycle catabolic process	3.900067476
nucleobase-containing compound catabolic process	3.638034376
nucleoside phosphate catabolic process	3.16227766
nucleotide catabolic process	3
organic cyclic compound catabolic process	4.146139914
organophosphate catabolic process	3.16227766
regulation of carbohydrate catabolic process	2
regulation of nucleotide catabolic process	2
ATP biosynthetic process	3
carbohydrate biosynthetic process	2.828427125
carbohydrate derivative biosynthetic process	3.356585567

© 2021. Jin C et al. Published online at http://www.aginganddisease.org/EN/10.14336/AD.2021.0520

SUPPLEMENTARY DATA

carboxylic acid biosynthetic process	3.605551275
coenzyme biosynthetic process	3
cofactor biosynthetic process	3.605551275
hexose biosynthetic process	2.236067977
monocarboxylic acid biosynthetic process	3.464101615
monosaccharide biosynthetic process	2.236067977
neurotransmitter biosynthetic process	2.236067977
nicotinamide nucleotide biosynthetic process	3
nitric oxide biosynthetic process	2.236067977
nucleobase-containing small molecule biosynthetic process	3.31662479
nucleoside monophosphate biosynthetic process	3
nucleoside phosphate biosynthetic process	3.16227766
nucleoside triphosphate biosynthetic process	3.16227766
nucleotide biosynthetic process	3.16227766
organic acid biosynthetic process	3.605551275
organophosphate biosynthetic process	3.356585567
purine nucleoside monophosphate biosynthetic process	3
purine nucleoside triphosphate biosynthetic process	3.16227766
purine nucleotide biosynthetic process	3.16227766
purine ribonucleoside monophosphate biosynthetic process	3
purine ribonucleoside triphosphate biosynthetic process	3.16227766
purine ribonucleotide biosynthetic process	3.16227766
purine-containing compound biosynthetic process	3.16227766
pyridine nucleotide biosynthetic process	3
pyridine-containing compound biosynthetic process	3
pyruvate biosynthetic process	3
reactive oxygen species biosynthetic process	2.645751311
regulation of ATP biosynthetic process	2
regulation of nitric oxide biosynthetic process	2
regulation of reactive oxygen species biosynthetic process	2.236067977
ribonucleoside monophosphate biosynthetic process	3
ribonucleoside triphosphate biosynthetic process	3.16227766
ribonucleotide biosynthetic process	3.16227766
ribose phosphate biosynthetic process	3.16227766
small molecule biosynthetic process	4.242640687
ATP generation from ADP	3
gluconeogenesis	2.236067977
glycolytic process	3
regulation of glycolytic process	2

© 2021. Jin C et al. Published online at http://www.aginganddisease.org/EN/10.14336/AD.2021.0520

SUPPLEMENTARY DATA

Cytokine secretion	
Terms	Z-score
cytokine production	5.567764363
cytokine secretion	3.31662479
negative regulation of cytokine production	3.31662479
negative regulation of tumor necrosis factor superfamily cytokine production	2
positive regulation of cytokine production	4.358898944
positive regulation of cytokine secretion	3
positive regulation of tumor necrosis factor superfamily cytokine production	2.645751311
regulation of cytokine production	5.291502622
regulation of cytokine secretion	3.16227766
regulation of tumor necrosis factor superfamily cytokine production	3.31662479
tumor necrosis factor superfamily cytokine production	3.464101615
interleukin-1 beta production	2.645751311
interleukin-1 beta secretion	2
interleukin-1 production	2.645751311
interleukin-1 secretion	2
negative regulation of tumor necrosis factor production	2
positive regulation of interleukin-1 beta production	2.236067977
positive regulation of interleukin-1 production	2.236067977
positive regulation of interleukin-6 production	2.236067977
positive regulation of tumor necrosis factor production	2.645751311
regulation of interleukin-1 beta production	2.449489743
regulation of interleukin-1 production	2.449489743
peptide secretion	4.146139914
positive regulation of peptide secretion	3.207134903
positive regulation of protein secretion	3.207134903
positive regulation of secretion	3.577708764
positive regulation of secretion by cell	3.299831646
protein secretion	4.146139914
regulation of peptide secretion	3.771236166
regulation of protein secretion	3.771236166
regulation of tumor necrosis factor production	3.31662479
tumor necrosis factor production	3.464101615
Chemotaxis	
Terms	Z-score
cell chemotaxis	3.872983346
chemotaxis	4.358898944
leukocyte chemotaxis	3.31662479

© 2021. Jin C et al. Published online at http://www.aginganddisease.org/EN/10.14336/AD.2021.0520

SUPPLEMENTARY DATA

macrophage chemotaxis	2.236067977
monocyte chemotaxis	2
positive regulation of chemotaxis	2.828427125
regulation of chemotaxis	3.16227766
ameboidal-type cell migration	3.050851079
blood vessel endothelial cell migration	2.449489743
endothelial cell migration	2.121320344
epithelial cell migration	2.529822128
epithelium migration	2.529822128
leukocyte migration	3.605551275
macrophage migration	2.449489743
mononuclear cell migration	2.449489743
myeloid leukocyte migration	3
positive regulation of blood vessel endothelial cell migration	2.236067977
positive regulation of cell migration	5.112077203
positive regulation of epithelial cell migration	2.121320344
positive regulation of leukocyte migration	2.645751311
regulation of blood vessel endothelial cell migration	2.449489743
regulation of endothelial cell migration	2.121320344
regulation of epithelial cell migration	2.529822128
regulation of leukocyte migration	2.828427125
regulation of macrophage migration	2
regulation of mononuclear cell migration	2
tissue migration	2.529822128
positive regulation of locomotion	5.112077203
positive regulation of cell motility	5.112077203
taxis	4.472135955
maintenance of location	3.356585567
positive regulation of cellular component movement	5.112077203
Immunity and inflammation	
Terms	Z-score
leukocyte activation involved in immune response	3
leukocyte degranulation	2.236067977
leukocyte mediated immunity	3.605551275
leukocyte proliferation	3.16227766
macrophage activation	2.236067977
mast cell activation	2.449489743
mast cell activation involved in immune response	2
mast cell degranulation	2

© 2021. Jin C et al. Published online at http://www.aginganddisease.org/EN/10.14336/AD.2021.0520

mast cell mediated immunity	2
mononuclear cell proliferation	3.16227766
myeloid cell activation involved in immune response	2.449489743
myeloid leukocyte activation	3.605551275
myeloid leukocyte mediated immunity	2.449489743
negative regulation of cell activation	3.050851079
negative regulation of immune system process	3.900067476
negative regulation of leukocyte activation	3.16227766
negative regulation of lymphocyte activation	2.645751311
cell activation involved in immune response	3
inflammatory response	4.041451884
neuroinflammatory response	2
positive regulation of defense response	2.886751346
regulation of defense response	3.130495168
regulation of leukocyte activation	3.638034376
regulation of mast cell activation	2
T cell activation	2.840187787
regulation of cell activation	3.709704134
Ion transport and homeostasis	
Terms	Z-score
calcium ion homeostasis	3
cation homeostasis	4.642383454
cellular calcium ion homeostasis	3
cellular cation homeostasis	4.535573676
cellular divalent inorganic cation homeostasis	3.299831646
cellular ion homeostasis	4.535573676
cellular iron ion homeostasis	2.645751311
cellular metal ion homeostasis	4.314554973
cellular transition metal ion homeostasis	3.16227766
divalent inorganic cation homeostasis	3.299831646
inorganic ion homeostasis	4.642383454
iron ion homeostasis	2.828427125
metal ion homeostasis	4.426352064
transition metal ion homeostasis	3.31662479
transition metal ion transport	2.449489743
regulation of cytosolic calcium ion concentration	2.713602101
positive regulation of cytosolic calcium ion concentration	2.529822128
Lysosome	
Terms	Z-score

© 2021. Jin C et al. Published online at http://www.aginganddisease.org/EN/10.14336/AD.2021.0520

lysosomal membrane	2.828427125
lysosome	4.426352064
lysosome localization	2.236067977
lytic vacuole	4.426352064
lytic vacuole membrane	2.828427125
late endosome	3.050851079
late endosome membrane	2
endosomal part	3
endosome membrane	3
vacuolar membrane	3.31662479
vacuolar part	3.31662479
vacuole	4.642383454
cytoplasmic vesicle part	3.050851079
Cellular response to stimulus	
Terms	Z-score
cellular response to inorganic substance	2.496150883
cellular response to metal ion	2.496150883
response to inorganic substance	3.837612894
response to metal ion	3
response to wounding	4.314554973
negative regulation of response to external stimulus	2.496150883
positive regulation of response to external stimulus	3.356585567
regulation of response to external stimulus	4.157609203
regulation of response to wounding	2.333333333
Angiogenesis	
Terms	Z-score
angiogenesis	3.299831646
blood vessel development	2.558408596
blood vessel morphogenesis	2.836832573
positive regulation of vasculature development	3.31662479
positive regulation of angiogenesis	3.31662479
vasculature development	2.710687383
regulation of vasculature development	2.496150883
regulation of angiogenesis	2.496150883
cardiovascular system development	2.710687383
Cell survival	
Terms	Z-score
negative regulation of neuron apoptotic process	2.121320344
neuron apoptotic process	3.050851079

© 2021. Jin C et al. Published online at http://www.aginganddisease.org/EN/10.14336/AD.2021.0520

regulation of neuron apoptotic process	2.713602101
negative regulation of neuron death	3.050851079
neuron death	3.577708764
regulation of neuron death	3.152963125
Protein-lipid complex	
Terms	Z-score
plasma lipoprotein particle	2
plasma lipoprotein particle organization	2
protein-lipid complex	2
protein-lipid complex subunit organization	2
lipoprotein particle	2
regulation of plasma lipoprotein particle levels	2.236067977

Supplementary Table 5. Statistics reporting.

Fig No.	Description	Group (n)	Test used	Degree of freedom and P value
Fig1b	Iba1+ cell body volume	Cortex: young(98) aged(89);<br>Striatum: young(103) aged(102);<br>SVZ: young(53) aged(82);<br>Hippocampus: young(65) aged(96)	Cortex: Unpaired t-test, two tailed;<br>Striatum: Unpaired t-test, two tailed;<br>SVZ: Unpaired t-test, two tailed;<br>Hippocampus: Mann Whitney test	Cortex: t (185)=-9.079,***p<0.001;<br>Striatum: t (161.683)=-9.542,***p<0.001;<br>SVZ: t(133)=-3.893,***p<0.001<br>Hippocampus: ***p<0.001;
Fig1c	Iba1+ cell number	Young (23): cortex (5), striatum (6), Hippocampus (6), SVZ (6);<br>Aged (24): cortex (6), striatum (6), Hippocampus (6), SVZ (6);	Mann Whitney test	*p=0.042
Fig2d	Proportion of subclusters	Young (8) aged (8)	Unpaired t-test, two tailed	C0:t(14)=1.321,p=0.208;<br>C1:t(14)=0.531,p=0.604<br>C2:t(14)=-1.244,p=0.234<br>C3:t(14)=-4.511,***p<0.001<br>C4:t(14)=2.734,*p=0.016<br>C5:t(14)=-3.361,**p=0.005
Fig2e	Markers of C0	C0 (1922) others (1931)	Wilcoxon rank sum test, Bonferroni correction	***p<0.001

© 2021. Jin C et al. Published online at http://www.aginganddisease.org/EN/10.14336/AD.2021.0520
